# Economic evaluations in undergraduate medical education: a systematic review

**DOI:** 10.1136/bmjopen-2024-091911

**Published:** 2025-03-13

**Authors:** Stijntje Willemijn Dijk, Skander Essafi, Christa Niehot, John B Wong, Myriam Hunink, Andrea M Woltman

**Affiliations:** 1 Epidemiology, Erasmus MC University Medical Center Rotterdam, Rotterdam, Zuid-Holland, Netherlands; 2 Radiology, Elisabeth-Tweesteden Ziekenhuis, Tilburg, Netherlands; 3 Radiology and Nuclear Medicine, Erasmus MC University Medical Center Rotterdam, Rotterdam, Zuid-Holland, Netherlands; 4 Erasmus University Rotterdam, Rotterdam, Zuid-Holland, Netherlands; 5 Medical Library, Erasmus MC University Medical Center Rotterdam, Rotterdam, Zuid-Holland, Netherlands; 6 Tufts Medical Center, Boston, Massachusetts, USA; 7 Center for Health Decision Sciences, Harvard T.H. Chan School of Public Health, Boston, Massachusetts, USA; 8 Institute for Medical Education Research Rotterdam, Erasmus MC University Medical Center Rotterdam, Rotterdam, Zuid-Holland, Netherlands

**Keywords:** MEDICAL EDUCATION & TRAINING, Health economics, Systematic Review

## Abstract

**Abstract:**

**Objectives:**

Medical education profoundly impacts patients, students, educators and public resources. However, the economic dimensions necessary for informed decision-making remain underexplored. This systematic review examines how economic evaluations are conducted and reported in undergraduate medical education (UME) literature and assesses their quality.

**Design:**

Systematic review.

**Data sources:**

Medline, Embase, Web of Science, Cochrane, ERIC, Google Scholar and the CEVR CEA databases were searched on 13 September 2024.

**Eligibility criteria:**

Eligible studies evaluated interventions within UME and reported (incremental) costs and effects, employing any method such as cost-effectiveness analysis, cost-minimisation analysis or decision-analytic modelling.

**Data extraction and synthesis:**

Key data, including study characteristics, evaluation type, perspective, intervention details, sensitivity analyses, cost and effect measures, outcomes, expressions of cost-effectiveness and adherence to economic reporting guidelines, were extracted. Quality was assessed using the CHEQUE tool, and the findings were synthesised qualitatively.

**Results:**

Of 6559 studies identified, 21 met the inclusion criteria. Most studies reported costs and effects post-hoc within effectiveness trials, with only one decision-analytic modelling study identified. Evaluated domains included instructional methods, skills training, selection and student health. All but one study adopted a payer (university) perspective, and nearly all focused on short-term outcomes. Sensitivity analyses were rarely performed, and no study achieved full quality scores.

**Conclusions:**

Economic evaluations in UME are scarce and often of limited methodological rigour. A shift towards comprehensive, prospective evaluations is needed to address long-term outcomes, societal perspectives and methodological robustness. Such efforts will enable better resource allocation, enhance the impact of medical education and contribute to a sustainable educational landscape.

**PROSPERO registration number:**

CRD42023478907.

**STRENGTHS AND LIMITATIONS OF THIS STUDY**

- This review applied a comprehensive and systematic search strategy developed in collaboration with an information specialist to identify relevant studies.
- The review incorporated a rigorous assessment of sensitivity analyses and uncertainty, addressing key methodological gaps in the included studies using the CHEQUE tool.
- The study included both trial-based evaluations and decision-analytic models, offering insights into diverse methodological approaches.
- The review was limited to undergraduate medical education, excluding potentially transferable findings from other areas of health professions education.

## Introduction

 Medical education shapes the future healthcare landscape by training competent and compassionate health professionals. The value of medical education extends beyond individual students; it has a profound impact on the healthcare system and society at large. The field is continuously evolving to equip healthcare professionals with the diverse competencies needed, requiring adaptations in both content and form to meet the dynamic challenges of the evolving healthcare landscape based on emerging demands, knowledge and technologies.

However, even if an educational intervention seems effective, it does not automatically mean that providing it is appropriate given the time and cost constraints.^1^ Efficiency, ensuring the greatest impact through cost-effectiveness with available resources, is one of the core principles of social accountability in medical schools.^2^ The concept of lean learning emphasises the reduction of inefficiency by eliminating unnecessarily complex and time-consuming practices, while directing resources to areas where they will have the greatest impact.^3^ In the past 60 years, the cost of medical education in the USA has increased by 750%.^4^ While much attention has been given to the effectiveness of educational interventions, another vital aspect—cost-effectiveness—remains underexplored in a world with increasing demands on educational institutions and finite resources.^5^

Effective educational innovations should undergo economic evaluations to verify their efficiency compared with alternative approaches across diverse contexts.^6^ Educators and policymakers who are considering a curricular change or other educational intervention need to know the potential benefits, risks, uncertainties, preferences, as well as the cost in order to take informed decisions.^7^ However, evaluations of costs are rare in medical education, ranging from 0% of publications evaluating undergraduate medical interventions in 1969 to 3% in 2007.^8^ A systematic review of e-learning interventions from 2014 concluded that of all 60 included papers, not one explicitly investigated cost-effectiveness, cost-benefit or cost utility.^9^

While previous authors have highlighted the need for the integration of economic evaluations in the area of medical education,^35 10^,^12^ full economic evaluations seem to be scarce. However, to the best of our knowledge, no systematic review of economic evaluations of the complete body of literature has been conducted. Related reviews have been conducted within specific types of interventions such as skills training, e-learning^913^,^15^ or continuous professional development,^16^ investigated cost-analysis without requiring trade-offs with effectiveness^12 15 17^ or had publication date restrictions.^12 17^ Neglecting to consider such trade-offs can lead to a suboptimal resource allocation, especially in cases where the effectiveness of two educational interventions is similar, but costs differ widely.

In response to this gap, our review aims to provide a comprehensive overview of how economic evaluations of undergraduate medical education (UME) are conducted, reported and how they align with quality standards for methods and reporting.

## Aim

We sought to answer the question: ‘How are economic evaluations conducted and reported in the field of UME?’; and we discuss the implications of these findings for the future of medical education and research. Our review focuses on interventions that seek to improve and enrich medical curriculum content, methodologies and the experiences of students or faculty within medical training programmes, or other aspects of medical education. The primary aim is to explore and present the landscape and methodology of decision-analytic models and economic evaluations within UME. Specifically, we review analyses that involve a trade-off between incremental benefits, such as improvements in students’ academic performance or the quality of life of patients, and incremental costs such as monetary costs and faculty time. The secondary aims were to investigate which methods were applied in UME cost-effectiveness analyses (CEAs), how included studies were performed considering health economic evaluation methods and reporting standards, which comparisons were made in which areas of UME, which cost and effect outcomes were estimated, how trade-offs in costs and effects were expressed and how uncertainty was handled in included papers.

## Methods

This review is reported according to the Preferred Reporting Items for Systematic Reviews and Meta-Analyses (PRISMA) guidelines for reporting systematic reviews.^18^ Table 1 provides an overview of definitions of key terms used throughout this manuscript.

**Table 1:** Glossary of economic terms used in this paper. Adapted from Dijk *et al*^69^

Glossary of terms	
Alongside trial CEA	A type of economic evaluation conducted concurrently with a clinical trial.
Cohort state transition model (Markov model)	A mathematical model that simulates the progression of a group of people through different states over time.
Consolidated Health Economic Evaluation Reporting Standards (CHEERS)	A set of guidelines designed to improve the reporting of health economic evaluations.
Cost analysis	A method that identifies, measures and compares the costs of different interventions.
Cost-effectiveness analysis (CEA)	A technique that compares the costs and health effects of two or more interventions.
Cost minimisation analysis	A type of economic evaluation used to compare the costs of two or more interventions that are considered to have equivalent effectiveness. In other words, it helps determine the least expensive way to achieve a specific health outcome.
Cost-effectiveness acceptability curve	A graphical representation of the probability that an intervention is cost-effective across a range of cost-effectiveness thresholds.
Criteria for Health Economic Quality Evaluation (CHEQUE)	A quality assessment tool for cost-effectiveness analyses.
Decision-analytic model	Decision-analytic models compare the expected costs and consequences of two or more decision options by combining the best available evidence from multiple sources and applying mathematical techniques.
Economic perspective	The viewpoint adopted to define the types of costs and benefits to consider in an economic evaluation. The choice of perspective determines which costs and benefits are included in the analysis and can significantly influence the results. The most common perspectives in health economic evaluations are the societal, healthcare and payer perspectives. The societal perspective considers all costs and benefits to society, including direct and indirect medical costs, productivity losses and quality of life. The healthcare perspective focuses on the costs and benefits incurred within the healthcare system, such as hospitals and medications. The payer perspective, such as a medical school, considers the costs and benefits from the viewpoint of a specific payer, for example, the costs of hiring additional faculty.
Incremental cost	The additional cost associated with one intervention compared with another.
Incremental Cost Effectiveness Ratio (ICER)	A ratio demonstrating the trade-offs between costs and benefits, calculated as the ratio of the incremental cost of an intervention to the incremental benefit in health outcomes.
Incremental effect	The additional health, learning or other benefit achieved by one intervention compared with another.
Incremental Net Health Benefit (iNHB)	A summary statistic representing the impact of an intervention on a population’s health for a given willingness-to-pay threshold, compared with an alternative intervention, calculated as:incremental health benefit−incremental cost of the intervention/willingness-to-pay threshold
Incremental Net Monetary Benefit (iNMB)	A summary statistic representing the value of an intervention in monetary terms for a given willingness-to-pay threshold, compared with an alternative intervention, calculated as:incremental health benefit*willingness-to-pay threshold–incremental cost of the intervention.
Net benefit	A summary statistic representing the impact of an intervention on a population outcome compared with an alternative intervention, calculated as either incremental net health benefit or incremental net monetary benefit.
Non-inferiority Randomised Controlled Trial	A clinical trial designed to demonstrate that a new treatment is not significantly worse than an established standard treatment.
Probabilistic analysis (PA)	A technique used to propagate uncertainty from model inputs to model outcomes. Also referred to in the literature as Probabilistic Sensitivity Analysis (PSA).
QALY	Quality-adjusted life year.
Sensitivity analysis	Sensitivity analysis is a technique used to understand how changes in input variables affect a specific output variable. It involves systematically varying the values of input variables to observe their impact on the output variable.
Value of information analysis (VOI)	The estimation of decision uncertainty and the value of collecting more information on key parameters influencing a decision, expressed in monetary or health terms.
Willingness to pay (WTP)	A threshold that represents what the decision maker or society is willing to pay for a unit of health outcome. The threshold is expressed in monetary units per health outcome.

### Eligibility criteria

To be eligible for inclusion, papers needed to meet the criteria listed in table 2.

**Table 2:** Inclusion and exclusion criteria according to PICOS

Item	Inclusion	Exclusion
Population	The study includes at least 20% undergraduate medical students (but can be a mix between, eg, medical students and residents or other health profession students). The study includes faculty involved in undergraduate medical education.	Only postgraduate/post-registration medical graduates (eg, residents). Students from other health professions. Community members/patients.
Intervention	Any intervention aimed at intervening within the system of medical education, the medical curriculum or other aspects of the medical school.	Interventions aimed to teach medical students about health economics and cost-efficiency. Interventions employing medical students as health workers to reduce costs in patient care (eg, employing students as medical scribes). Interventions that take place outside of the medical school setting or that do not address aspects of medical education, medical student life or the medical school setting.
Comparator	Any comparator. A comparison must be made in order to report incremental costs and benefits.	No comparator (eg, cost-analysis or a description of an intervention).
Outcome	Any reported incremental cost (eg, monetary cost or time invested by faculty) and effect (eg, quality-adjusted life years or academic performance) that is numerically quantified.	Qualitative descriptions of costs or cost-effectiveness (eg, staff considered the intervention cost-effective). Analyses that only report costs, or only report effects, or only report these for the intervention arm.
Study type	Economic evaluations: cost-utility analysis, cost-benefit analysis, cost-minimalisation analysis. Decision-analytic models. Alongside trial cost-effectiveness analysis.	Not an economic evaluation. Cost analysis (no effects). Opinion papers, reviews, letters to the editor, conference abstracts. Not available in English.

We included studies that evaluated interventions targeting UME students and faculty. These interventions aimed to improve aspects of medical education (systems), such as curriculum design, teaching methods or student health. We focused on studies that compared different interventions and reported both costs (monetary or time) and effects (eg, student performance, patient outcomes). Economic evaluations, including cost utility, cost-benefit and cost-minimisation analyses, as well as decision-analytic models, were eligible for inclusion. Decision-analytic models compare the expected costs and consequences of two or more decision options by combining the best available evidence from multiple sources and applying mathematical techniques.^19^

Studies that did not involve a comparison group, relied solely on qualitative assessments of costs or effects, or were not reported in English were excluded. Studies without a comparison group were excluded because they do not meet the criteria for a full economic evaluation, which requires a comparison of costs and effects between two or more alternatives. Non-English studies were excluded due to resource constraints and the need to ensure consistency in interpretation among the research team.

### Information sources and search strategy

A search strategy was developed by an information specialist (CN) in cooperation with the lead author (SWD). The search was developed in Embase.com, optimised^20^ for sensitivity and then translated to other databases. The search was carried out in the databases Medline, Embase, Web of Science, Cochrane and ERIC. Additionally, a search was performed in Google Scholar from which the 200 highest-ranked references were downloaded using the software Publish or Perish^21^ and the CEVR CEA database. The search contained terms for (1) medical students and (2) costs and cost analysis. No study registries were searched, but Cochrane CENTRAL retrieves the contents of ClinicalTrials.gov and WHO’s International Clinical Trials Registry Platform. Systematic reviews identified by our search were screened for additional citations that met our inclusion criteria.

The full searches for each database are described in online supplemental table 1.

### Selection and data collection process

The information specialist (CN) eliminated duplicate search results using Endnote^22^ and imported identified papers into Rayyan QCRI,^23^ a freely available software for in- and excluding papers in systematic reviews. Data extraction was performed in Microsoft Excel.

Two reviewers (SWD and SE) assessed papers for inclusion and data extraction. Disagreements were resolved by consensus by SWD and SE or by the full research team.

### Data items

Extracted data items include study characteristics, type of economic evaluation, study sample, medical education domain, economic perspective, intervention, comparator, cost measure, outcome measure, cost, outcome, willingness-to-pay WTP) threshold, sensitivity analyses, the authors’ conclusions on cost-effectiveness and the stated use of reporting guidelines for reporting economic evaluations, such as the currently the most widely used guideline Consolidated Health Economic Evaluation Reporting Standards (CHEERS).^24^ When papers did not explicitly state which strategy was the intervention and which the comparator, we reported the strategy that was presented as more novel as the intervention.

### Study quality assessment

We assessed the quality of including studies using the Criteria for Health Economic Quality Evaluation (CHEQUE tool).^25 26^ Our assessment focused on the methodological and reporting quality of the economic evaluation within the selected papers, rather than the risk of bias of, for example, the trial that they are based on.

We generated a concise visual representation of the assessment using statistical software R^27^ similar to the Risk of Bias visualisations from the package *robvis*.^28^ The total scores are computed using CHEQUE’s score weighting system. We provide both the weighted scores where we assign a full score for items valued as N/A (not applicable), and where we exclude N/A values from both the numerator and denominator. An attribute is not applicable, for example, if a question addresses a modelling aspect, but the assessed study is not a decision-analytic model. The final scores were expressed as a percentage of the maximum obtainable score.

### Cost and effect outcomes

We report costs and effects as incremental. All costs were converted into US$2023 using an exchange rate and inflation calculator.^29^

### Synthesis methods

Within the review synthesis, we first reflect on the overall methodological considerations of included papers, followed by individual study cost-effectiveness results, types of economic evaluations, educational domains, expression of cost-effectiveness and sensitivity analyses and handling of uncertainty. Within types of economic evaluation, we distinguish economic evaluations alongside trials^30^ from economic evaluations that synthesise data from disparate sources using decision-analytic models.^19^

## Results

### Study selection

**Figure 1:**
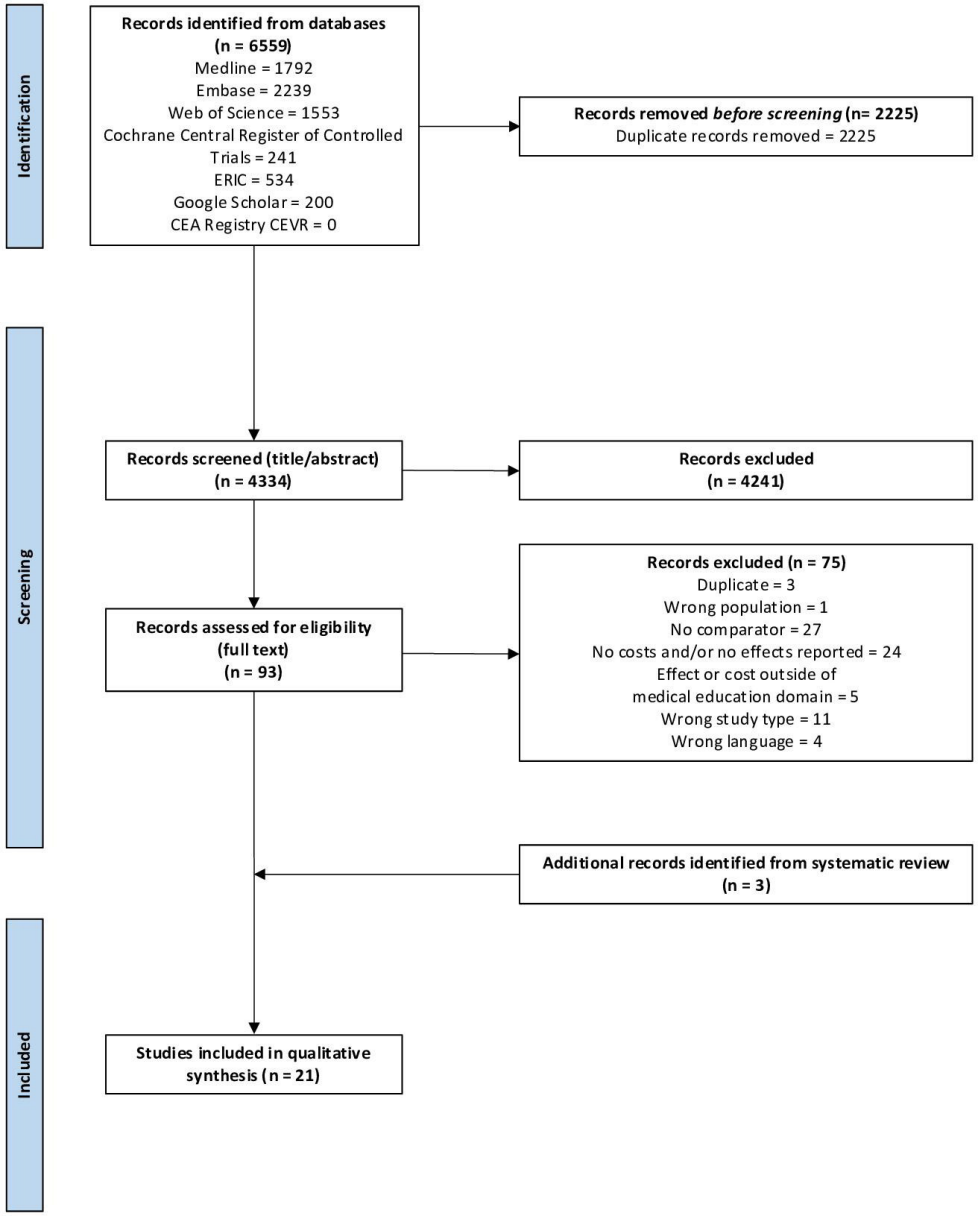
PRISMA flowchart. PRISMA, Preferred Reporting Items for Systematic Reviews and Meta-Analyses.

Our search conducted on 13 September 13 2024 identified 6559 papers. ([Supplementary-material SP1]Online supplemental table 2[Supplementary-material SP1]) After eliminating duplicates, we excluded 4241 papers based on title and abstract. 93 papers underwent an evaluation of their eligibility through full-text assessment, resulting in the exclusion of 75 papers. The reasons for exclusion included duplication (n=3, 4%), insufficient representation of medical students (n=1, 1%), absence of a comparator (n=27, 36%), lack of reported costs and/or effects (n=24, 32%), effects and/or costs beyond the scope of UME (n=5, 7%), incorrect study type (n=11, 15%) or non-availability in English (n=4, 5%). We identified three additional papers from systematic reviews.^31^,^33^ Ultimately, 21 met the inclusion criteria for the systematic review.^31^,^51^ Figure 1 represents a visual representation of this process through a PRISMA flowchart.

### Study characteristics

Table 3 provides a comprehensive overview of the included study characteristics (extended version in online supplemental table 3). Studies were conducted in the USA, UK, Canada, Netherlands, Australia and Germany (online supplemental figure 1), and were published between 1994 and 2021 (online supplemental figure 2). The study designs of selected articles were randomised controlled trials (n=12, 57%), non-randomised trials (n=4, 19%) and cross-sectional comparisons, case studies or cohort studies (n=4). We identified only one decision-analytic model (n=1, 5%).^48^ Among the range of economic evaluation approaches were (alongside trial) CEAs and cost-minimisation studies, one study used a case study combined with a net benefit regression analysis and one was a cost-benefit analysis. In other cases, the authors did not explicitly investigate cost-effectiveness, but simply reported costs and effects. The economic perspectives adopted in the studies were all from a payer (university) perspective, with only one study considering a societal perspective.^48^ Outcome measures were multifaceted, encompassing assessments of knowledge acquisition and skills proficiency (n=20, 95%), student satisfaction (n=8, 38%) and student health (n=1, 5%). Additionally, the studies employed mostly monetary cost measures, with the exception of one study using staff allocation (full-time equivalents; FTEs) per student,^49^ and one study using man-hours^38^ as a cost measure.

**Table 3:** Study characteristics for the systematic review on economic evaluations in medical education

Author, year, country	Type of economic evaluation	Medical education domain	Intervention	Comparator 1	Comparator 2	Outcome measure
Allen, 2011, USA^51^	Cost-minimisation analysis	Consultation skills	Patient educators for physical examination teaching	Physician-educators	N/A	OSCE performance
Bandla, 2012, USA^36^	Alongside trial CEA	General instructional approaches	e-learning	Face-to-face learning	N/A	Level 1: Learner satisfaction; Level 2: Knowledge of sleep medicine; Level 3: Application or transfer of behaviour to practice
Bosse, 2015, Germany^38^	Alongside trial CEA	Consultation skills	Peer roleplay	Standardised patients	N/A	OSCE of six stations addressing challenging parent–physician interactions with global rating scales ranging from 100=completely agree to 1=strongly disagree
Chandrasekera, 2006, UK^37^	Alongside trial CEA	Practical skills training	Cardboard box skills training	‘Conventional’ Pelvic trainer skills training	N/A	Score of transfer tasks and time taken for each task. Scores and times calculated in three domains (cube transfer, mint transfer, disc cut out) for dominant and non-dominant hand
De Giovanni, 2009, Canada^33^	Alongside trial CEA	Practical skills training	High-fidelity heart sound simulator (Harvey)	CD heart sounds training	N/A	Communication and examination skills scores
Ford, 2016, UK	Cost-minimisation analysis	General instructional approaches	Group simulated ward round	Individualised feedback	N/A	Student evaluationStudent performance on patient safety in an OSCE
Hasle, 1994, USA^50^	Cost-minimisation analysis	Consultation skills	Patient educator-led physical diagnosis sessions	Physician-led physical diagnosis sessions	N/A	Student performance (OSCE score)
Hauer, 2009, USA^31^	Alongside trial CEA	Consultation skills	Web-based standardised patient examination	In-person formative standardised patient examination	N/A	Scores on a subsequent high stake standardised patient examinationSatisfaction
Isaranuwatchai, 2013, Canada	Net benefit regression model	Practical skills training	(PROGRESS) mid-fidelity programme (inanimate plastic arm)	Low-fidelity (LOW) computer-based simulator programme	High-fidelity (HIGH) programme (human patient simulator)	Direct Observation of Procedural Skills (DOPS)
Janjua, 2018, UK^45^	Alongside trial CEA	Practical skills training	Gynaecological Pelvic Examination on expert patients (Gynaecological Teaching Associates, GTAs)	Traditional teaching using manikins (TARGET)	N/A	Assessment of confidence and competence in performing pelvic examinations
Nieuwenhuijzen Kruseman, 1997, Netherlands^49^	CEA	General instructional approaches	Problem-based learning	Relatively less problem-based learning	N/A	OSCE results, Student satisfaction
Lemke, 2020, Canada^41^	Alongside trial cost minimisation analysis	Practical skills training	Holography augmented (HA) for suture training	Faculty-led (FL) suturing training	Peer-tutor led (PTL) suturing training	Nr. of interrupted sutures placed to achieve proficiency; Nr. of full-length (75 cm) sutures used to achieve proficiency; Time to proficiency (minutes); Student preference; Student confidence
Maloney, 2015, Australia^35^	Alongside trial CEA	General instructional approaches	BL: Blended learning (10 two-hour face-to-face classes with additional activities and mobile learning)	F2F: Traditional learning (10 two-hour face-to-face classes)	N/A	Evidence-based medicine competency; Quality-adjusted students educated (QASE), using the formula QASE=number of students educated x the group’s average rating on the Berlin Questionnaire
Matsumoto, 2002, Canada^46^	Alongside trial CEA	Practical skills training	Hands-on training using endourological bench models: low fidelity	Hands-on training using endourological bench models: high fidelity	Didactic session	Endourological performance
McDougall, 2009, USA^32^	Alongside trial CEA	Practical skills training	Didactic session with video demonstration of laparoscopic suturing+virtual reality simulator	Didactic session with video demonstration of laparoscopic suturing+silicone model and pelvic trainer	N/A	Objective structured assessment of technical skills of laparoscopic cystography
Nathan, 2021, UK	Alongside trial CEA	General instructional approaches	Virtual classroom training (VCT)	Face-to-face training (FFT)	Non-interactive computer-based learning (CBL)	Post-intervention Objective Structured Assessment of Technical Skills score
Rosenthal 2009, USA^43^	Cost minimisation analysis	Practical skills training	Southwestern video trainer station (SW) and Fundamentals of laparoscopy (FLS)	Proficiency-based FLS only	N/A	Number of repetitions to reach required proficiency
Schreurs, 2018, Netherlands^39^	Cost-benefit analysis	Student selection	Multimethod selection	Lottery	N/A	Monetary savings based on dropouts, repetition of blocks, repetition of OSCEs
Smith, 1997, USA^48^	CEA	Student health	Hepatitis A vaccination for all students	Serotesting and vaccinating the seronegative against hepatitis A	N/A	QALYs
Stefanidis, 2009, USA	Alongside trial CEA	Practical skills training	Basic unsupervised laparoscopic skills training+tutorial video+suturing	No basic training+tutorial video+suturing	N/A	Skill proficiency and retention
Taylor, 2013, UK^42^	Alongside trial CEA	General instructional approaches	Station-based group: comment on how a student performed on each task	Skills-based group: give students an overall rating of how they did on generic skills	Both	OSCE scoreStudent satisfaction

CEA, cost-effectiveness analysis; N/A, not applicableOSCE, Objective Structured Clinical Examination; QALY, quality-adjusted life year

### Quality assessment of studies

One study mentions the CHEERS statement.^39^ No other papers explicitly refer to economics-based guidelines in their reports.

**Figure 2:**
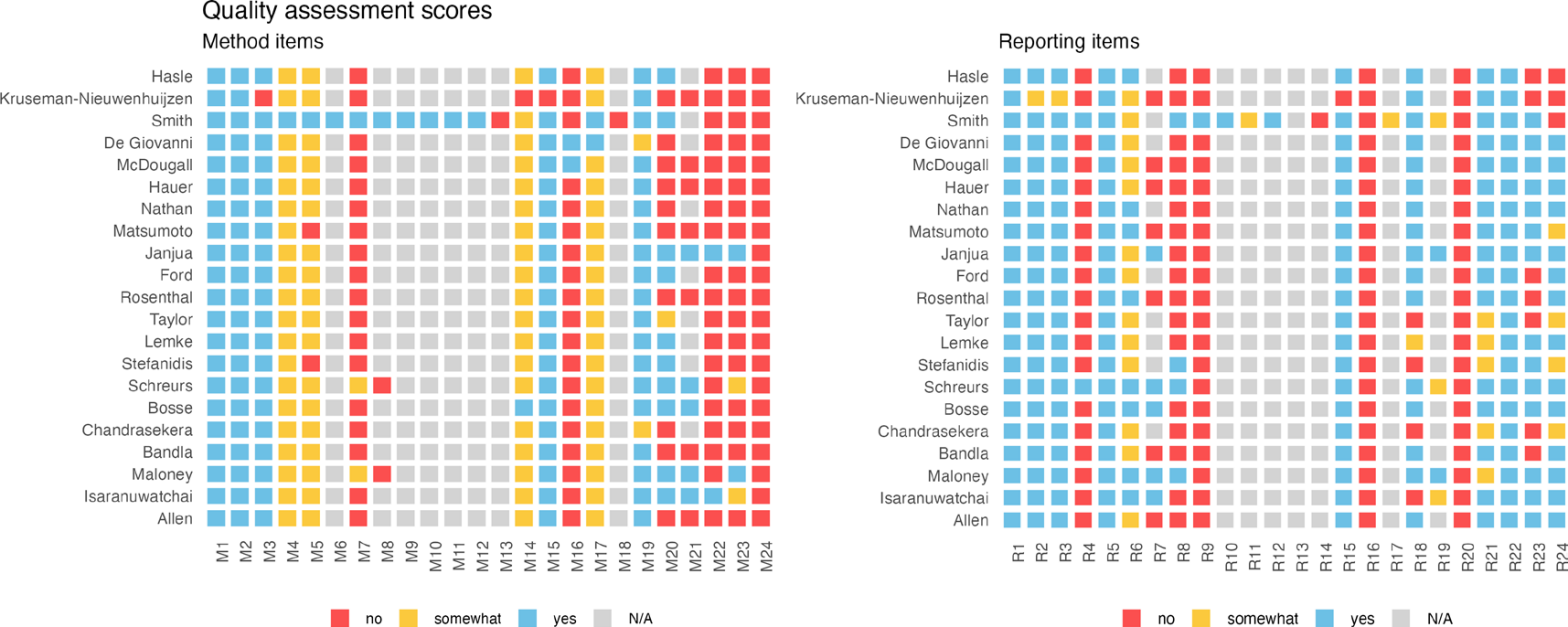
Quality assessment scores for individual studies based on CHEQUE, separated across methods and reporting attributes. CHEQUE, Criteria for Health Economic Quality Evaluation.

Figure 2 illustrates the individual study scores for each method and reporting item from the CHEQUE tool. The mean weighted quality assessment for methods*,* expressed as the percentage of the maximum attainable percentage score was 55% (range: 18%–70%%) when excluding N/A-rated items and 72% (range: 55%–81%) when assigning full scores to N/A rated items. For reporting, these scores were 73% (range: 28%–72%) and 82% (range: 63%–94%). No study obtained a full score.

Most studies scored high on relevance, specification of goals and future implications to decision making, referencing source data. However, there were several items where no or few studies received full scores. For example, no studies addressed equity considerations or comprehensively summarised potential consequences in an impact inventory table. No study reported attempts to validate their results. Papers rarely explicitly assessed the quality of their data or sources of bias (n=2, 10%). Few studies explored alternative assumptions and scenarios (n=3, 14%) or did so partially (n=2, 10%). Two studies (10%) performed probabilistic (sensitivity) analysis, which is used to account for the uncertainty in multiple parameters, and constructed a cost-effectiveness acceptability curve. Such curves show the probability that an intervention is cost-effective across a range of WTP thresholds. Several items on methodology and reporting were only applicable to one model-based study and assigned N/A in other cases. A detailed report of all considerations towards the quality assessment using CHEQUE in this study is published in Dijk *et al*.^26^

### Individual study results

**Figure 3:**
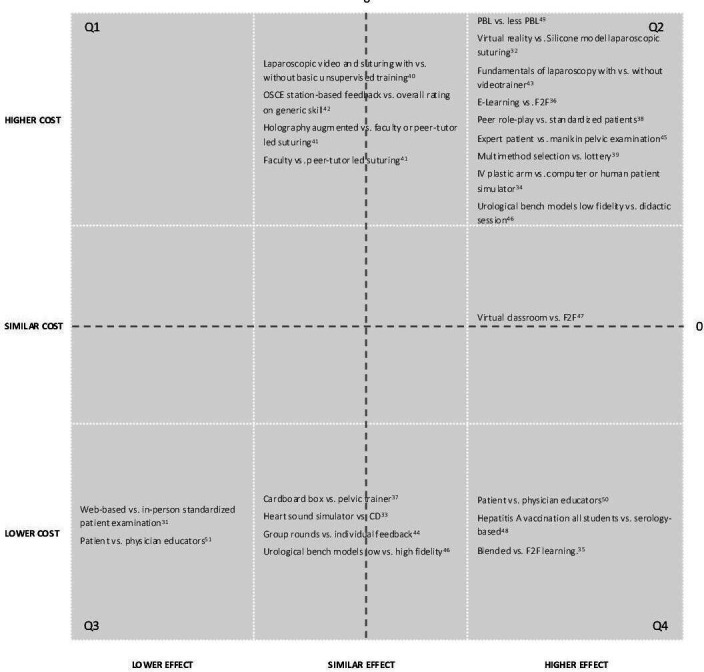
Descriptive cost-effectiveness plane. On the Y-axis, we show the categories higher, similar and lower costs of the intervention (first mentioned) versus the comparator on the X-axis. Interventions falling within the right lower quadrants (Q4: higher/similar effect and lower/similar cost) should be implemented, and those in the left upper quadrant (Q1: lower/similar effect and higher/similar cost). The remaining items (Q2/Q3) require a trade-off between the additional costs or cost-savings and the additional effects gained or foregone, which depends on the willingness-to-pay and the available resources. The numbers in the image refer to the references.

The results of individual studies are summarised in table 4, figure 3 and online supplemental table. When multiple outcomes were mentioned, in most cases no primary outcome was selected, leading to multiple and sometimes conflicting conclusions on cost-effectiveness. Not all papers explicitly stated which was the intervention and which was the comparator strategy. While a WTP threshold is needed to state whether an intervention is cost-effective when there is a trade-off, no study explicitly chose such a threshold. Two studies^34 45^ (10%) did perform a sensitivity analysis in which they assessed cost-effectiveness across a range of WTP values (online supplemental table).

**Table 4:** Effectiveness and economic outcomes of the systematic review of economic evaluations in medical education

Author	Included in costs	Incremental cost *	Effect outcome	Cost-effectiveness expression	WTP threshold	Sensitivity analyses	Conclusions on cost-effectiveness
Allen	Faculty hours, educators, physicians, time	−$342†	Incremental student performance: −1.1%, p=0.0279	Cost and effects reported separately	N/R	N/R	Lower cost and slightly lower effect
Bandla	Faculty cost, supplies purchased (CD-ROMs, video production)	+$148†	Level 1: Incremental effect: Instruction effectiveness −0.39, application of sleep knowledge −0.27; Level 2: Incremental effect 6%; Level 3: Incremental OSCE score 0.6, Clerkship 0.03	Cost and effects reported separately	N/R	N/R	Higher cost and higher effect on level 2 and 3
Bosse	Personnel time	−46.4% of personnel hours	3.6% improved OSCE score	ICER=+0.74 for communication training with student roleplays+0.45 for standardised patients	N/R	N/R	Higher cost and higher effect
Chandrasekera	Material cost	$46 482	Sugar cube transfer: in cardboard box trainer assessment=+1 in pelvic trainer assessment=+2Disc cut out: in cardboard box trainer assessment=+3; in pelvic trainer assessment=0	Cost and effects reported separately	N/R	N/R	Lower cost and similar effect
De Giovanni	Materials	$106 336	Identification of sounds: −0.64 (p=0.06); Diagnosis: −0.10; Communication skills: 0.7; Examination skills: 0.1	Costs and effects reported separately	N/R	N/R	Higher cost and similar effect
Ford	Volunteer patients, travel expenses, catering, clinical skills consumables, staff	−$72.36†	No difference in overall OSCE performance	Costs and effects reported separately	N/R	N/R	Lower cost and similar effect
Hasle	Standardised patients, education specialists, faculty adviser, supplies, director	−$44 952.82	2% better OSCE performance	Costs and effects reported separately	N/R	N/R	Lower cost and higher effect
Hauer	SP time, production cost, additional staff	−$4229	Incremental Performance score=−0.18Incremental Satisfaction=−0.38	Costs and effects reported separately	N/R	N/R	Lower cost and similar to lower effect
Isaranuwatchai	Materials, personnel, maintenance, consumables	Mid-fidelity versus low-fidelity: +$65 701Mid-fidelity versus high-fidelity: +$15 570	Incremental procedural skills scores: Mid-fidelity versus low-fidelity: +15; Mid-fidelity versus high-fidelity: +6.52	iNB across various WTP thresholds	$0–$100 000	$0–$100 000 WTP; All versus only implementation cost; Cost-effectiveness acceptability curve	Higher cost and higher effect compared with both low and high fidelity.
Janjua	Materials, personnel	$59.2†	12 more students were considered competent at pass level and 28 more students competent at merit and distinction levels	ICER=+$1044 per competent student and +$447 per student competent at merit level	$0–$2600/extra% competent student	One-way sensitivity analysis on cost	Higher cost and higher effect
Kruseman-Nieuwen-huijzen	Personnel, faculty development	82.6 FTEs	Performance in clinical skills: +12% in satisfactory OSCE itemsKnowledge skills: +7% in correct minus incorrect answersStudent satisfaction: +0.4	Costs and effects reported separately	N/R	N/R	Higher cost and higher effect
Lemke	Personnel, materials	HA versus FL: $39.26†HA versus PTL: $54.39†FL versus PLT: $15.13†	Respectively for FL, PTL, HA: Nr. of simple interrupted sutures placed to achieve proficiency: 80, 85, 105.4Nr. of full-length (75 cm) sutures used to achieve proficiency: 9.3, 10.3, 11Time to proficiency (minutes): 158, 162.2, 205 (p=0.390)Student preference: 100%, 80%, 8.3%	Costs and effects reported separately	N/R	N/R	HA versus FL and PTL: Higher costs and similar effectFL versus PTL: Higher cost and similar effect
Maloney	Personnel, preparation personnel, space	Fixed cost transition to BL: $31 225; Variable cost: −$10 232; Overall difference of −85 personnel hours	Incremental effect of +77 QASE	ICER: −$0.86/QASE	N/R	Scenario analysis around increased transition costs and staffing requirements for BL	Lower cost and higher effect
Matsumoto	Bench models, staff examiners	Incremental to didactic session: Low fidelity model cost of production +$23 High fidelity model cost: +$4236	Low fidelity versus didactic sessions: Global rating scale: +7.6; Checklist score: +9.5; Pass rating: +11.5High-fidelity similar scores compared with didactic sessions (exact change not reported)	Costs and effects reported separately	N/R	N/R	Low versus High fidelity bench model: Lower cost and similar effect Low fidelity versus didactic sessions: Higher cost and higher effect
McDougall	Materials, maintenance, personnel	VR laparoscopic simulator: +$113 622; Pelvic trainer: +$32 609; Maintenance of the simulation range from +$11 400 to $21 500Incremental cost: $102 316	Incremental Objective Structured Assessment: 0.6 (p=0.24)	Costs and effects reported separately	N/R	N/R	Higher cost and higher effect
Nathan	Suturing equipment, technology, venue	VCT to FFT: +$25.77†VCT to CBL: +$25.77†	VCT was non-inferior to FFT (adjusted difference: 0.44), VCT superior to CBL (1.69) and FFT superior to CBL (1.25)	Costs and effects reported separately	N/R	N/R	VCT versus FFT and CBL: Similar cost and higher effect
Rosenthal	Consumable materials, time	+$399	−13.5 repetitions	Costs and effects reported separately	N/R	N/R	Higher cost and higher effect
Schreurs	Personnel, assessment, dropout	+$42 726	Total benefits of selection (in dropout and repetition of blocks monetary value): +€206 745	Costs and effects reported separately	N/R	N/R	Higher cost and higher effect
Smith	Cases of hepatitis care, Vaccines, Serology, Time off work	−$22 781	+0.6 QALYs (Not reported, but calculated from the ICER and incremental cost)	ICER=-$34 400/QALY	N/R	Threshold analyses	Lower cost and higher effect
Stefanidis	Materials and time	+$210†	−29 repetitions for the basic training group −165 min training time for the basic training group	Costs and effects reported separately	N/R	N/R	Higher cost and similar effect
Taylor	Personnel	−$3.33†	OSCE scores were respectively for skills-based feedback and station-based feedback 65.6 and 65.9 (part 1 subjects) then 70 and 70.5 (part 2 subjects).	Costs and effects reported separately	N/R	N/R	Higher cost and similar effect

* If costs were expressed as monetary cost, these were converted to US$2023 USD. All costs are shown as intervention versus comparator. ***: Costs per student. Study characteristics are described in Table 2. Controlled Trial.

† Costs per student. Study characteristics are described in table 3.

BL, blended learning; CBL, computer-based learning; CEA, cost-effectiveness analysis; F2F, face to face; FFTface to face trainingFL, faculty led; FLS, fundamentals of laparoscopy; GTA, Gynaecological Teaching Associates; HA, holography augmented; HIGH, high fidelity; ICER, Incremental Cost-Effectiveness Ratio; LOWlow fidelityN/A, not applicable; N/R, not reported; OSCE, Objective Structured Clinical Examination; PTL, peer-tutor led; QALY, quality-adjusted life year; QASE, quality-adjusted students educated; SP, simulated patient; VCT, virtual classroom training

Most comparisons in figure 3 had interventions with higher effects and higher costs, requiring a trade-off by the institution to consider whether the benefit outweighs the additional cost. However, there were also interventions with lower costs and higher or similar effects, suggesting implementation without trade-off.

### Types of economic evaluation

#### Decision-analytic models

The study by Smith *et al* from 1997^48^ was the only model-based study identified by our review. The study is a Markov model in which a hypothetical cohort of medical students is followed across their lifetime to estimate the number of hepatitis A cases and lost (quality-adjusted) years of life with serological screening prior to vaccination, versus vaccinating all students. Throughout the model, students are in one of four possible health states: susceptible, immune, acute hepatitis A infection or dead. The model estimated that without vaccination, there would be 286 hepatitis A cases with 4 deaths and 107 lost years of life over the lifetimes of 66 629 students enrolled in medical school in the USA in 1997. Vaccination of all students was cost-effective compared with serological screening.

#### Other analysis types

In those studies that conducted CEAs alongside a trial, results were presented as the immediate costs and effects of the trial itself, usually without any extrapolation to alternative situations or towards the future. Maloney *et al*^35^ did report their results across a 10-year time period, considering differences in fixed upfront costs and long-term costs of face-to-face and blended learning. Furthermore, cost-minimisation analysis was used for studies in which the explicit objective was to ascertain the same effect, but at a lower cost.^41 43 44 50 51^ Some minimisation studies did not explicitly state that their objective was cost-minimisation but do mention this in the conclusion.^51^

One study (Schreurs *et al*, 2018)^39^ performed a cost-benefit analysis, in which both costs and effects are expressed in monetary terms. In their study, the authors converted the benefits of less student dropout, less repetition of blocks and less repetition of Objective Structured Clinical Examinations (OSCEs) to Euros for their sample and extrapolated the costs and benefits to the full cohort.

Isaranuwatchai *et al*^34^ studied a case in which 15 students used one of three catheterisation skills programmes (low-fidelity, high-fidelity or a progressive combination) and then used a net benefit regression analysis to identify which programme was cost-effective. For each participant, they calculated the Net Benefit (NB) using the equation NB=WTP*E–C, where E represents the effect and C the cost, across a range of WTP values between $0 and $100 000. They then constructed a regression model based on the NB, interventions and potential confounding factors (sex, education, training and practice).

### Educational domains

Authors discussed UME areas including general instructional approaches (n=6), practical skills training (n=9), consultation skills training (n=4), student selection (n=1) and student health (n=1).

*General instructional approaches* compared digital and face-to-face learning, or instructional methods such as blended and problem-based learning (PBL) on student knowledge and satisfaction, or the time and manner in which students received feedback. A key factor influencing the cost-effectiveness of a digital approach was the inclusion of which costs across which time period. For example, when two instructional cases were blended and considered only immediate costs, digital deliveries were more expensive.^36 47^ However, the study by Maloney *et al* included not only immediate costs but also long-term costs.^35^ After 3 years, blended learning was saving costs compared with face-to-face learning costs as the costs of creating content were no longer in place, and the number of hours in direct teaching was lower. Nieuwenhuijzen-Kruseman *et al*,^49^ who reviewed the cost-effectiveness of PBL, compared their staff allocation to the other medical schools in the Netherlands and assumed differences in general student performance (the effectiveness) were due to differential PBL-hour allotments.

Authors who reviewed *practical skills training interventions* (eg, suturing, laparoscopy, IV placement or pelvic examination) compared novel technologies such as simulators, holography augmentation and virtual reality to more traditional methods such as manikins and CDs, as well as low-cost alternatives such as cardboard boxes. Notably, despite significant cost discrepancies in some interventions (eg, a heart sound simulator’s incremental cost of approximately $75 000), innovative high-cost interventions were reported as having similar effects on student performance to simpler interventions such as the use of CD heart sounds.^33^

The studies in which *consultation skills* were trained compared patient-, peer-led and physician-led teaching to either maximise physical examination and communication skills, or to minimise cost.

In the paper by Schreurs *et al*,^39^ the authors investigated the costs and (monetary) benefits of a multi-method *medical school selection* process versus a lottery admittance system. While the lottery system incurred negligible costs to the medical school, the average benefits of multimethod selection were much higher with reduced dropout, reduced repetition of courses and reduced examination resits across the 3-year medical bachelor.

Finally, the study by Smith *et al*^48^ on Hepatitis A vaccination (described under subsection ‘Decision analytic models’) was the only study that investigated *student health*.

### Cost domains

One study^52^ uses a specific guideline^53^ for reporting cost for clinical examination. Most studies included personnel costs and materials. Rarely were costs separated into fixed and variable costs, and rarely was cost-effectiveness investigated across a time horizon beyond immediate results. The costs of maintenance were not negligible for interventions, such as the high-fidelity heart sound simulator, but were not included in the cost-effectiveness comparison.^33^ One study included the cost of student dropout,^39^ and one study the costs related to cases of hepatitis care and future time off work.^48^

We did not identify any studies that incorporated the costs of, for example, infrastructure, costs borne by learners such as time, travel and opportunity costs or costs and benefits to patients and society at large.

### Expression of cost-effectiveness trade-off

Some studies presented an Incremental Cost Effectiveness Ratio (ICER), which is calculated as the incremental cost of the new intervention divided by the incremental benefit. When no trade-off is necessary and one of the interventions is dominant (has both higher effect and lower costs), an ICER does not need to be calculated. Other studies did not explicitly express cost-effectiveness, but rather presented the costs and effects separately within the paper. One study expressed cost-effectiveness in terms of NB.^34^

### Sensitivity analyses and uncertainty

Three papers investigated alternative choices and assumptions through sensitivity and scenario analyses. These studies explored various scenarios, including what would happen if the interest rate changes,^45^ if the authors included the costs of training teaching associates,^45^ what would happen if not the full cohort but only half the cohort received the intervention,^39^ or what if transition costs or staffing requirements increased.^35^

Janjua *et al*^45^ used one-dimensional bootstrapping to establish confidence intervals around variables such as cost. While all except two papers focused on deterministic analyses, Janjua *et al*^45^ also applied Probabilistic (Sensitivity) Analysis. In this approach, they used two-dimensional bootstrapping, in which they resampled costs and outcomes simultaneously to generate a distribution on a cost-effectiveness plane. Moreover, Isaranuwatchai *et al* and Janjua *et al*^34 45^ extended their analysis by generating a Cost-Effectiveness Acceptability Curve (CEAC). This curve offers decision-makers a tool to assess the probability that an intervention is cost-effective compared with its counterpart across various WTP values.

## Discussion

In this review, we explored the landscape of economic evaluations in UME, shedding light on both observed practice and gaps in the literature. Our review highlights the limited quantity and quality of economic evaluations in UME. We found that: (a) economic evaluations in UME are scarce and of limited methodological rigour, with most studies reporting costs and effects post-hoc rather than incorporating economic considerations prospectively; (b) nearly all studies focused narrowly on immediate payer costs and short-term effects, omitting broader societal perspectives, long-term outcomes and equity considerations; (c) sensitivity analyses and uncertainty assessments were rarely performed, with most studies relying on deterministic methods that limit the robustness of conclusions and (d) while interventions often required a trade-off between costs and benefits, few studies explicitly quantified these trade-offs using frameworks such as Incremental Cost-Effectiveness Ratios (ICERs) or CEACs. These findings underscore the need for more comprehensive, prospective and methodologically sound economic evaluations in UME to guide policy decisions effectively.

Our review highlights a discrepancy between the growing recognition that evidence-based medical education should address constraints on time and resources,^10^ and the application of such analyses. While previous authors called for more economic evaluations^35 10^,^12^ and even provided guidance on how to conduct^5^ or assess^11^ published literature in this field, the full body of UME literature had not been previously reviewed. Our review emphasises the necessity to shift towards more comprehensive evaluations that recognise the complexity of educational interventions, their uncertainty and their broader societal impact to contribute to a more sustainable resource allocation and a more impactful education landscape.

With finite resources available, the principles of social accountability in medical schools require us to aim for the best value for (often public) money.^2 54^ Decision-making requires a careful consideration of available information, the likelihood of a range of possible outcomes and societal values.^7^ Most decisions by policymakers bring along an opportunity cost: the resources used for one intervention could also be invested elsewhere. This requires priority-setting. Failing to consider trade-offs between costs and effects in UME leads to a suboptimal allocation of finite resources. This consideration is especially important in cases where the effectiveness of two educational interventions is similar, but costs differ widely.

Many core principles from economic guidance were not or only partially implemented. Of the studies that we did include in this review, the majority only stated costs and effects after a trial or cross-sectional analysis was conducted, whereas proper economic evaluations require consideration upfront.^30^ While no one type of analysis (eg, cost-effectiveness analysis or cost-minimisation analysis) is universally more appropriate in medical education contexts, the choice of economic evaluation method should align with the specific research question being addressed.

Previous authors attributed the scarcity of economic evaluations in medical education to the prevalence of qualitative research methods over quantitative approaches^55^ and a lack of reliable tools for assessing educational effectiveness.^56^ Historically, most evaluations in UME focus on relatively ‘low’ levels of outcomes such as learner satisfaction or (self-reported) gain in skill, rather than deep cognitive, psycho-motor, emotional or behavioural changes,^57^ patient outcomes, or doing justice to the wider tasks of education to enable a diverse group of students to develop themselves as future health professionals^58^ while preserving their well-being. This observation was also reflected by our study; while such evaluations are beneficial for internal quality assurance, their limited scope offers less valuable insights into discussions on long-term value^57^ and cost-effectiveness. Nearly all studies included in our review considered only costs and effects immediately after intervention. Cost-effectiveness is likely to be largely underestimated when omitting the costs of providing poor quality medical education, such as the consequences of medical errors.^3 59 60^

The scope of identified studies was also narrow, where nearly all only considered a payer perspective and included personnel and material costs, but not long-term and societal consequences. Additionally, more frequent applications of trials in decision-analytic models could offer new opportunities to explore expected long-term and broader impact of educational interventions. One such decision-analytic model was published after our search date and was therefore not included in our review, but can serve as an example for future reference.^61^ An overview of common types of models and what questions they could answer in the area of UME is provided in table 5.

**Table 5:** Common decision-analytic model types, descriptions and examples applied in the field of medical education

Model type	Description	Example application
Decision tree	Simple yet powerful, decision trees calculate expected values by weighing chances and outcomes. They assist in making choices by visualising the potential consequences of different strategies and can be used to calculate the expected value of each overall strategy.	When a university is faced with choosing between software packages, a decision tree can help assess the trade-offs between upfront costs, maintenance and risks.
State-transition cohort model (Markov model)	These models track sequences of events over time, recurring for hypothetical cohorts. They are invaluable for understanding long-term implications and trends within a defined cohort.	In deciding whether to implement a mindfulness-based stress intervention programme for medical students, a state transition model follows a hypothetical cohort from enrolment to graduation and looks at how quality of life and student dropout are affected with and without the programme.^61^ ^66^
State-transition microsimulation model	Microsimulation models track the progression of simulated individuals rather than cohorts. This allows for a nuanced exploration of an interventions’ impact on diverse individuals.	A microsimulation model could help the university understand how certain career supportive measures could be allocated to specific (groups of) faculty to reduce inequalities.
Discrete event simulation	Focusing on unrestricted time periods, discrete event simulations map the progress of individuals through systems. They can help identify bottlenecks and evaluate the consequences of procedural adjustments.	Researchers could use discrete event simulation to enhance the efficiency of Objective Structured Clinical Examinations, by investigating the potential impacts of adjustments in factors like station numbers and personnel.
Dynamic model	Interactivity plays a central role in dynamic models, where individuals interact within a system. These models are ideal for studying the dynamic effects of interventions within a population.	A university-wide model considers all students and faculty, which uses dynamic modelling to compare COVID-19 prevention strategies such as mask-wearing and changes in class size.
Agent-based model	In addition to the interaction between individuals (=agents) within a model, their individual behaviour is also modelled.	A simulation of the regulations for social distancing and mask-wearing that takes into account how individuals make choices in the face of these measures.

Multiple quality items from the CHEQUE tool were not or rarely addressed appropriately, or addressed at all in included papers. None of the included studies addressed equity considerations or consequences for decision making, whereas these could be especially important in matters of student selection,^62^ assessment,^63^ patient involvement^2 64^ and efforts to reduce time and monetary costs to students. Additionally, we had set out to provide a comprehensive overview of all aspects of costs that were included in economic evaluations in medical education, but found that these were commonly limited to personnel and materials. We could also not provide guidance on the use of WTP levels as none of the included studies selected one.

While the importance of uncertainty is recognised as one of the underpinnings of medicine and evidence-based medical (education) research, uncertainty was rarely considered by included studies. Sensitivity analyses can assess the robustness of the conclusions of any analysis over a range of structural assumptions, probability estimates or outcome values.^7^ To be confident about a decision, one should explore how assumptions will affect a decision in a ‘what if’ analysis.^7^ Costs specifically were reported as a single number with no room for consideration of errors. Only a handful of papers performed some form of sensitivity analysis where the impact of specific scenarios or the combined uncertainty of costs and effects was investigated. To deal with uncertainties in a given decision problem, one should seek the best available evidence to support or refute their assumptions and use a framework for combining all of these uncertainties into a coherent choice.^7^ The reported costs and effects in individual studies and in this review should therefore be read with caution. Decision-analytic models, combined with probabilistic sensitivity analysis and value of information analysis, could offer the medical education field methods of extrapolating results beyond the trials, incorporating uncertainty and quantifying the consequences of making a decision or collecting more evidence.^65^ ^66^ As these types of models were not previously covered in the existing guidance or reviews in UME specifically, this paper provides an overview of example applications and model types (table 5).

### Strengths and limitations

Strengths of our review include the comprehensiveness of our search and our exploration of economic evaluations in medical education, which contribute to building knowledge in a previously underexplored topic. We did not apply restrictions to publication year or a specific domain of UME. By reviewing comparable papers together, some interesting patterns were observed, such as that many high-cost skills-based interventions did not result in higher effects. The two papers^51 60^ identified by a related systematic review and that were also eligible according to our inclusion criteria were also identified by our review, supporting the thoroughness of our search. We also apply the newly developed CHEQUE health economic evaluation quality assessment tool in a systematic review setting for the first time.^26^ This results both in a thorough assessment of the quality of included economic evaluations, as well as contributes to the further development of the CHEQUE tool itself^26^

However, our review also has limitations. Some relevant papers could be missed if they were not identified as cost-effectiveness analyses or did not imply within the abstract that both costs and effects were measured. Three additional papers from systematic reviews were identified for which this was the case. Additionally, our decision to focus solely on UME would likely have excluded papers that universal aspects applicable from other fields of higher (health professions) education,^16 67^ although cost-effectiveness research is also limited in other areas of higher education.^68^ In summarising costs and estimates, we only provided absolute numbers without uncertainty to support readability. Additionally, we had intended to provide a usable overview of the various cost domains considered by individual papers, but found little variation in what was included (such as personnel and material costs). The overview previously compiled from papers included in the cost-review by Yaros *et al*^12^ and the book by Walsh *et al*^3^ provides better guidance to future researchers in this respect than our compilation in table 4.^12^ Lastly, we excluded many papers without an explicit comparator (n=27), as we considered analysis of cost-effectiveness requires a comparison between two alternatives. We would have identified more studies if we had also included studies with pre-intervention and post-intervention tests, calculated the cost of educating a student, or considered the status quo the comparator. We did not consider papers that implicitly assumed a strategy with null costs and null effects as a comparator without explicit evaluation, as this approach may lead to an underestimation of the true implications of the status quo.

## Conclusion

In conclusion, our study not only advances the current discourse on economic evaluations in UME, but also sheds light on practical considerations relevant to everyone invested in the future of healthcare. The implications underscore the need for a shift towards comprehensive evaluations, recognising the complexity of educational interventions and their broader consequences. Future research should prioritise holistic, long-term context-specific approaches to cost-effectiveness analyses in medical education that address uncertainty and societal consequences. In doing so, we collectively contribute to a more sustainable and impactful medical education landscape.

Cost-effectiveness should not merely be the concern of economists; it is a matter of relevance to every individual who cares about the trajectory of medical education and, by extension, the future of healthcare. Each dollar expended can only be spent once, underscoring the need to ensure its allocation in a manner that maximises impact while minimising the burden on students and the broader society that bears the cost.

## supplementary material

10.1136/bmjopen-2024-091911 online supplemental file 1

## Data Availability

All data relevant to the study are included in the article or uploaded as supplementary information.
