# Supplementary material for: Economic evaluations in undergraduate medical education: a systematic review
Source: BMJ Open. 2025 Mar 13;15(3):e091911. doi: 10.1136/bmjopen-2024-091911 (PMC11907045; doi:10.1136/bmjopen-2024-091911)
Supplement: online supplemental file 1 [file bmjopen-15-3-s001.docx]

# Supplemental materials

## Search Strategy

| MEDLINE | ("Students, Medical"/ OR (((medicine* OR medical*) ADJ3 (student* OR curricul* OR facult* OR teach*)) OR undergraduate*).ab,ti,kf. OR ((("Education, Medical"/ AND (model OR models).ab,ti,kf.) OR (((medicine* OR medical*) ADJ3 (educat*) ADJ3 (model OR models))).ab,ti,kf.))) AND ("Cost-Benefit Analysis"/ OR "Cost-Effectiveness Analysis"/ OR exp "Costs and Cost Analysis"/ OR (((cost*) ADJ3 (analys* OR effect* OR utilit*)) OR cost*-and-benefit* OR ((economic*) ADJ3 (evaluat* OR value*)) OR ((decision*) ADJ2 (analytic*) ADJ2 (model*))).ab,ti,kf.) NOT (news OR congres* OR abstract* OR book* OR chapter* OR dissertation abstract*).pt. |
| --- | --- |
| EMBASE | ('medical student'/de OR (((medicine* OR medical*) NEAR/3 (student* OR curricul* OR facult* OR teach*)) OR undergraduate*):ab,ti,kw OR ((('medical education'/de AND (model OR models):ab,ti,kw) OR (((medicine* OR medical*) NEAR/3 (educat*) NEAR/3 (model OR models))):ab,ti,kw))) AND ('cost benefit analysis'/exp OR 'cost benefit model'/exp OR 'economic evaluation'/de OR 'cost effectiveness analysis'/exp OR 'cost utility analysis'/exp OR 'cost minimization analysis'/exp OR (((cost*) NEAR/3 (analys* OR effect* OR utilit*)) OR cost*-and-benefit* OR ((economic*) NEAR/3 (evaluat* OR value*)) OR ((decision*) NEAR/2 (analytic*) NEAR/2 (model*))):ab,ti,kw) NOT ([Conference Abstract]/lim OR [preprint]/lim) |
| Web of Science | TS=((((medicine* OR medical*) NEAR/2 (student* OR curricul* OR facult* OR teach*)) OR undergraduate*) OR (((medicine* OR medical*) NEAR/2 (educat*) NEAR/2 (model OR models)))) AND TS=(((cost*) NEAR/2 (analys* OR effect* OR utilit*)) OR cost*-and-benefit* OR ((economic*) NEAR/2 (evaluat* OR value*)) OR ((decision*) NEAR/2 (analytic*) NEAR/2 (model*))) NOT DT=(Meeting Abstract OR Meeting Summary) |
| Cochrane CENTRAL | ((((medicine* OR medical*) NEAR/3 (student* OR curricul* OR facult* OR teach*)) OR undergraduate*):ab,ti,kw OR (((medicine* OR medical*) NEAR/3 (educat*) NEAR/3 (model OR models))):ab,ti,kw) **AND** ((((cost*) NEAR/3 (analys* OR effect* OR utilit*)) OR cost* NEXT/1 "and" NEXT/1 benefit* OR ((economic*) NEAR/3 (evaluat* OR value*)) OR ((decision*) NEAR/2 (analytic*) NEAR/2 (model*))):ab,ti,kw) |
| ERIC | (medical students/ OR (((medicine* OR medical*) ADJ3 (student* OR curricul* OR facult* OR teach*)) OR undergraduate*).ab,ti. OR (((medical education/ AND (model OR models).ab,ti.) OR (((medicine* OR medical*) ADJ3 (educat*) ADJ3 (model OR models))).ab,ti.))) AND ("cost effectiveness"/ OR (((cost*) ADJ3 (analys* OR effect* OR utilit*)) OR cost*-and-benefit* OR ((economic*) ADJ3 (evaluat* OR value*)) OR ((decision*) ADJ2 (analytic*) ADJ2 (model*))).ab,ti.) NOT (news OR congres* OR abstract* OR book* OR chapter* OR dissertation abstract*).pt. |
| Google Scholar | "medicine\|medical student\|students\|curriculum\|curricula\|faculty\|teacher\|teachers\|teaching" cost\|costs\|economic analysis\|utility\|value\|"analytic model"\|effectiveness  'medicine\|medical student\|students\|curriculum\|curricula\|faculty\|teacher\|teachers\|teaching' cost\|costs\|economic analysis\|utility\|value\|'analytic model'\|effectiveness |
| CEVR  CEA registry | Abstract is: medical student or Abstract is: medical education or Abstract is: faculty or Abstract is: curriculum |

Supplementary Table 1: Search Strategy

## Search results

| Database searched | Platform | Years of coverage | Records | Records after duplicates removed |
| --- | --- | --- | --- | --- |
| Medline ALL | Ovid | 1946 - Present | 1792 | 1792 |
| Embase | Embase.com | 1971 - Present | 2239 | 1240 |
| Web of Science Core Collection* | Web of Knowledge | 1975 - Present | 1553 | 682 |
| Cochrane Central Register of Controlled Trials | Wiley | 1992 - Present | 241 | 107 |
| ERIC | Ovid | 1965 - Present | 534 | 383 |
| CEA Registry | CEVR | 1976 - Present | 0 | 0 |
| Additional Search Engines: Google Scholar** | | | 200 | 130 |
| Total | | | **6559** | **4334** |

Supplementary Table 2: Search results

*Science Citation Index Expanded (1975-present) ; Social Sciences Citation Index (1975-present) ; Arts & Humanities Citation Index (1975-present) ; Conference Proceedings Citation Index- Science (1990-present) ; Conference Proceedings Citation Index- Social Science & Humanities (1990-present) ; Emerging Sources Citation Index (2005-present)
**Google Scholar was searched via "Publish or Perish" to download the results in EndNote.

No other database limits were used than those specified in the search strategies

| Author, Year, Country | Study type | Type of economic evaluation | Medical Education domain | Study Sample | Economic perspective | Intervention | Comparator 1 | Comparator 2 | Time horizon | Economic evaluation reporting guideline | Outcome measure |
| --- | --- | --- | --- | --- | --- | --- | --- | --- | --- | --- | --- |
| Allen, 2011, USA | Non-randomized trial with historic control | Cost-minimization analysis | Consultation skills | 527 Second year medical students | Payer | Patient educators for physical examination teaching | Physician-educators | N/A | Immediate | N/R | OSCE performance |
| Bandla, 2012, USA | Non-randomized trial with historic control | Alongside trial CEA | General instructional approaches | 173 Third-year medical students on pediatrics clerkship | Payer | e-learning | Face to Face learning | N/A | Immediate | N/R | Level 1: Learner satisfaction  Level 2: Knowledge of Sleep Medicine  Level 3: Application or transfer of behavior to practice |
| Bosse, 2015, Germany | RCT | Alongside trial CEA | Consultation skills | 69 Fifth year medical students | Payer | Peer roleplay | Standardized patients | N/A | Immediate | N/R | OSCE of six stations addressing challenging parent-physician interactions with global rating scales ranging from 100 = completely agree to 1 = strongly disagree |
| Chandrasekera, 2006, UK | RCT | Alongside trial CEA | Practical skills training | 36 Third-year medical students | Payer | Cardboard box skills training | ‘Conventional’ Pelvic trainer skills training | N/A | Immediate | N/R | Score of transfer tasks and time taken for each task. Scores and times calculated in three domains (cube transfer, mint transfer, disc cut out) for dominant and non-dominant hand |
| De Giovanni, 2009, Canada | RCT | Alongside trial CEA | Practical skills training | 37 Third year medical students | Payer | High fidelity heart sound simulator (Harvey) | CD heart sounds training | N/A | Immediate | N/R | Communication and examination skills scores |
| Ford, 2016, UK | Cross-sectional study | Cost-minimization analysis | General instructional approaches | 23 Final year medical students | Payer | Group simulated ward round | Individualized feedback | N/A | Immediate | N/R | Student evaluation  Student performance on patient safety in an OSCE |
| Hasle, 1994, USA | Non-randomized trial with historic control | Cost-minimization analysis | Consultation skills | 17 Second year medical students | Payer | Patient educator-led physical diagnosis sessions | Physician-led physical diagnosis sessions | N/A | Immediate | N/R | Student performance (OSCE score) |
| Hauer, 2009, USA | RCT | Alongside trial CEA | Consultation skills | 150 Third year medical students | Payer | Web-based standardized patient examination | In-person formative standardized patient examination | N/A | Immediate | N/R | Scores on a subsequent high stake standardized patient examination  Satisfaction |
| Isaranuwatchai, 2013, Canada | Cross-sectional study | Net benefit regression model | Practical skills training | 35 Medical students with experience in peripheral intravenous (IV) catheters ‘starts’ | Payer | (PROGRESS) mid-fidelity program (inanimate plastic arm) | Low-fidelity (LOW) computer-based simulator program | High-fidelity (HIGH) program (human patient simulator) | Immediate | N/R | Direct Observation of Procedural Skills (DOPS) |
| Janjua, 2018, UK | RCT | Alongside trial CEA | Practical skills training | 418 Final year medical students | Payer (Faculty) | Gynecological Pelvic Examination on expert patients (Gynecological Teaching Associates, GTAs) | Traditional teaching using manikins  (TARGET) | N/A | Immediate | N/R | Assessment of confidence and competence in performing pelvic examinations |
| Nieuwenhuijzen Kruseman, 1997, Netherlands | Cross-sectional study | CEA | General instructional approaches | 25-250 Medical students | Payer | Problem based learning | Relatively less problem-based learning | N/A | Immediate | N/R | OSCE results, Student satisfaction |
| Lemke, 2020, Canada | RCT | Alongside trial cost minimization analysis | Practical skills training | 44 Second year medical students | Payer | Holography augmented (HA) for suture training | Faculty-led (FL) suturing training | Peer-tutor led (PTL) suturing training | Immediate | N/R | -Number of simple interrupted sutures placed to achieve proficiency  -Number of full-length (75 cm) sutures used to achieve  proficiency  -Time to proficiency (minutes)  -Student preference  -Student confidence |
| Maloney, 2015, Australia | RCT | Alongside trial CEA | General instructional approaches | 497 Third-year medical students | Payer | BL: Blended learning (ten 2-hour face-to-face classes with additional activities and mobile learning) | F2F: Traditional learning (ten 2-hour face-to-face classes) | N/A | Results reported across 10 years | N/R | Evidence based medicine competency  Quality-adjusted students educated (QASE), using the formula QASE = number of students educated x the group’s average rating on the Berlin Questionnaire |
| Matsumoto, 2002, Canada | RCT | Alongside trial CEA | Practical skills training | 40 Final year medical students | Payer | Hands-on training using endourological bench models: low fidelity | Hands-on training using endourological bench models: high fidelity | Didactic session | Immediate | N/R | Endourological performance |
| McDougall, 2009, USA | RCT | Alongside trial CEA | Practical skills training | 20 Laparoscopically naive medical students | Payer | Didactic session with video demonstration of laparoscopic suturing + virtual reality simulator | Didactic session with video demonstration of laparoscopic suturing + silicone model and pelvic trainer | N/A | Immediate | N/R | Objective structured assessment of technical skills of laparoscopic cystography |
| Nathan, 2021, UK | Non-inferiority RCT | Alongside trial CEA | General instructional approaches | 24 Medical students from all years | Payer | Virtual classroom training (VCT) | Face-to-face training (FFT) | Non-interactive computer-based learning (CBL) | Immediate | N/R | Post-intervention Objective Structured Assessment of Technical Skills score |
| Rosenthal 2009, USA | Non-randomized trial with historic control | Cost minimization analysis | Practical skills training | 20 second year medical students | Payer | Southwestern video trainer station (SW) and Fundamentals of laparoscopy (FLS) | Proficiency-based FLS only | N/A | Immediate | N/R | Number of repetitions to required reach proficiency |
| Schreurs, 2018, Netherlands | Longitudinal cohort | Cost-benefit analysis | Student selection | 286 medical students year 1-3 (Bachelor) | Payer | Multimethod selection | Lottery | N/A | 3 years | CHEERS | Monetary savings based on dropouts, repetition of blocks, repetition of OSCEs |
| Smith, 1997, USA | Cohort state transition model (Markov Model) | CEA | Student health | All 66,629 Medical students in the US in 1997 | Societal | Hepatitis A vaccination for all students | Serotesting and vaccinating the seronegative against hepatitis A | N/A | Lifetime | N/R | QALYs |
| Stefanidis, 2009, USA | RCT | Alongside trial CEA | Practical skills training | 20 Second year medical students | Payer | Basic unsupervised laparoscopic skills training + tutorial video+ suturing | No basic training + tutorial video + suturing | N/A | Immediate | N/R | Skill proficiency and retention |
| Taylor, 2013, UK | RCT | Alongside trial CEA | General instructional approaches | 350 Final year medical students | Payer | Station-based group: comment on how a  student performed on each task | Skills-based group: give students an overall rating of how they did on generic skill | Both | Immediate | N/R | OSCE score  Student satisfaction |

Supplementary table 3: Extended table - Study characteristics for the systematic review on economic evaluations in medical education. RCT = Randomized Controlled Trial. N/A = Not Applicable. N/R = Not reported. CEA = Cost-Effectiveness-Analysis. OSCE = Objective Structured Clinical Examination. QALY = Quality Adjusted Life Year. FLS = Fundamentals of laparoscopy. CBL = computer-based learning. GTA = Gynecological Teaching Associates. FL = Faculty led. HA = Holography augmented. LOW = low fidelity. HIGH = high fidelity. F2F = Face to face. QASE = Quality-adjusted students educated. PTL = Peer-tutor led. VCT = Virtual classroom training, FFT = Face to Face Training.

## Global distribution of studies


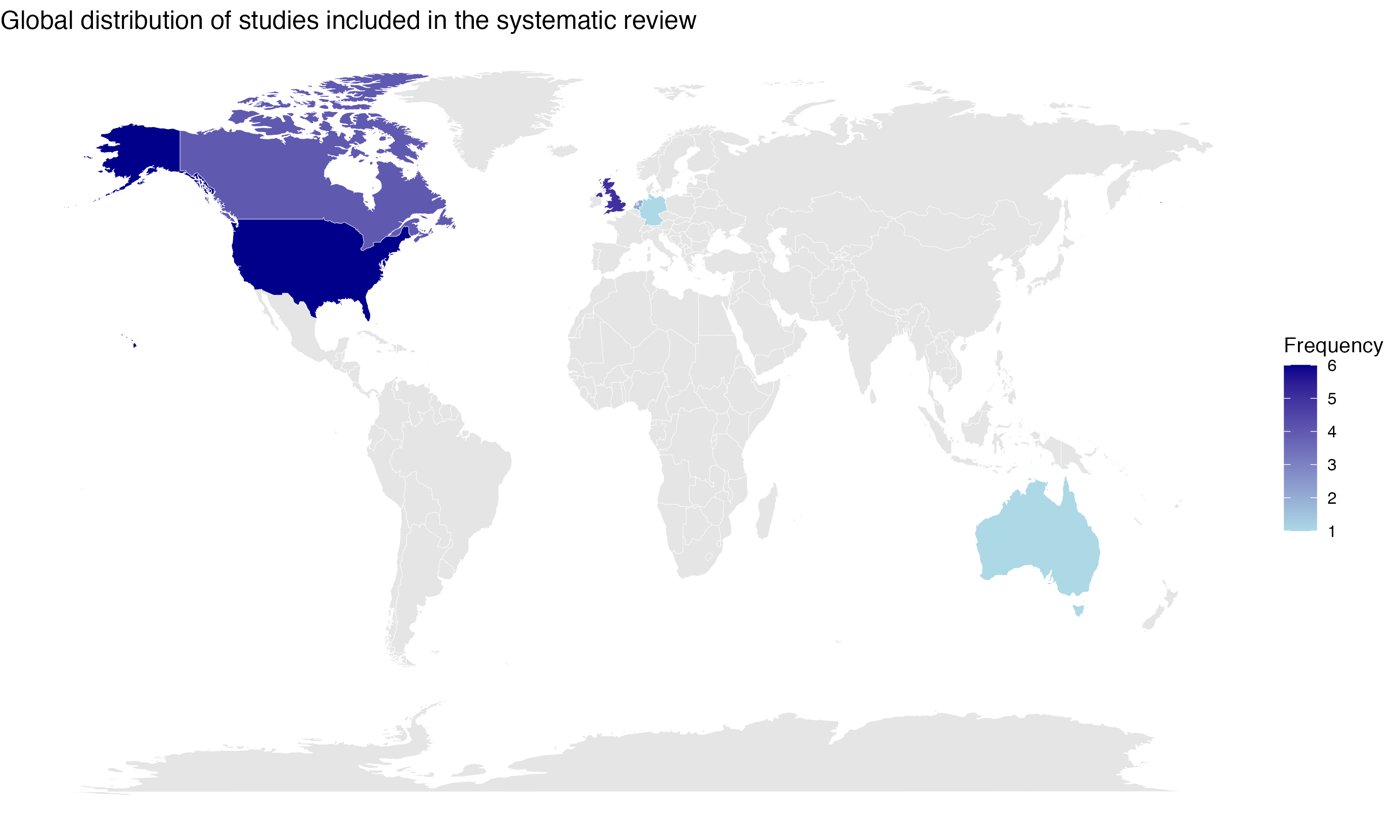


Supplementary figure 1: Global distribution of studies. Included studies were limited to the USA, Canada, Australia, UK, Netherlands, and Germany. In the full text screening stage, 4 studies were excluded as they could not be assessed by the authors based on non-English language. These studies were based in Switzerland, Germany (2) and Norway. One study that was excluded as fewer than 20% of participants were medical students but was otherwise eligible was based in Lebanon.

## Publications time-trend


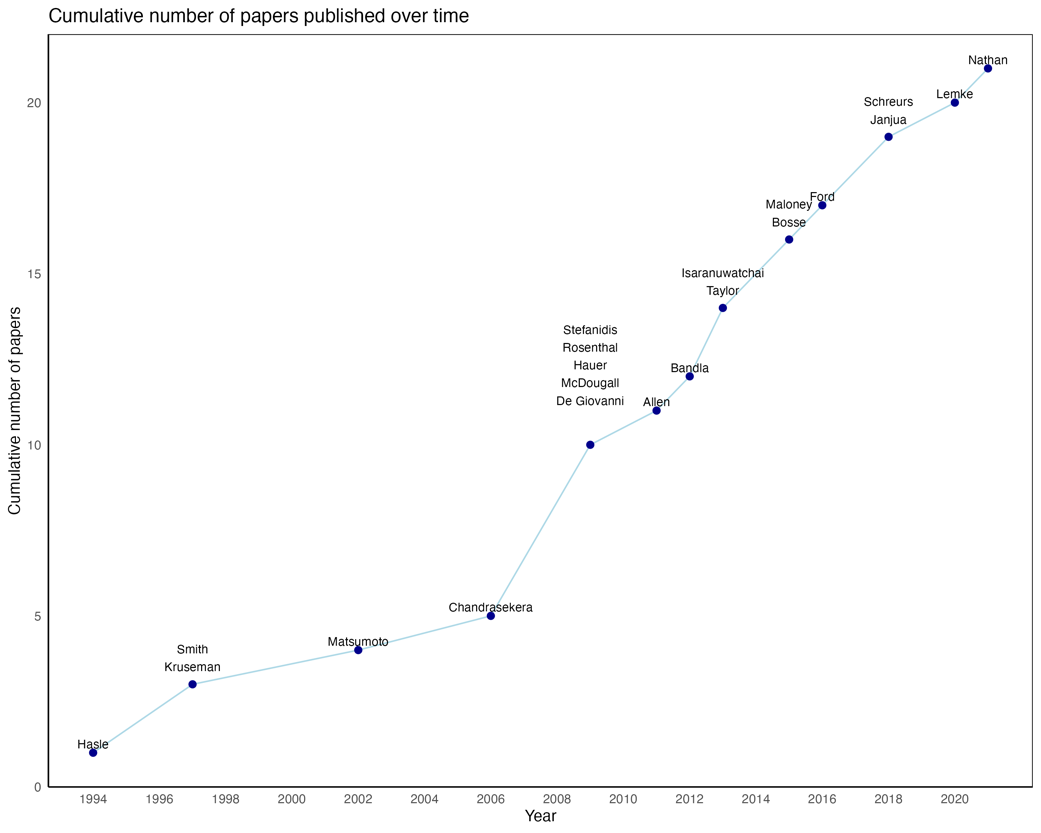


Supplementary figure 2: Cumulative number of papers published over time. Included papers were published between 1994 and 2021. The increase in the number of published papers is relatively stable over time, with most papers published after 2009.

## PRISMA statement

| **Section and Topic** | **Item #** | **Checklist item** | Location where item is reported |
| --- | --- | --- | --- |
| **TITLE** | | |  |
| Title | 1 | Identify the report as a systematic review. | Title |
| **ABSTRACT** | | |  |
| Abstract | 2 | See the PRISMA 2020 for Abstracts checklist. | Abstract |
| **INTRODUCTION** | | |  |
| Rationale | 3 | Describe the rationale for the review in the context of existing knowledge. | Introduction |
| Objectives | 4 | Provide an explicit statement of the objective(s) or question(s) the review addresses. | Aim |
| **METHODS** | | |  |
| Eligibility criteria | 5 | Specify the inclusion and exclusion criteria for the review and how studies were grouped for the syntheses. | Table 1 |
| Information sources | 6 | Specify all databases, registers, websites, organizations, reference lists and other sources searched or consulted to identify studies. Specify the date when each source was last searched or consulted. | Information Sources and Search Strategy |
| Search strategy | 7 | Present the full search strategies for all databases, registers and websites, including any filters and limits used. | Information Sources and Search Strategy |
| Selection process | 8 | Specify the methods used to decide whether a study met the inclusion criteria of the review, including how many reviewers screened each record and each report retrieved, whether they worked independently, and if applicable, details of automation tools used in the process. | Selection and data collection process |
| Data collection process | 9 | Specify the methods used to collect data from reports, including how many reviewers collected data from each report, whether they worked independently, any processes for obtaining or confirming data from study investigators, and if applicable, details of automation tools used in the process. | Selection and data collection process |
| Data items | 10a | List and define all outcomes for which data were sought. Specify whether all results that were compatible with each outcome domain in each study were sought (e.g. for all measures, time points, analyses), and if not, the methods used to decide which results to collect. | Data items |
|  | 10b | List and define all other variables for which data were sought (e.g. participant and intervention characteristics, funding sources). Describe any assumptions made about any missing or unclear information. | Data items |
| Study risk of bias assessment | 11 | Specify the methods used to assess risk of bias in the included studies, including details of the tool(s) used, how many reviewers assessed each study and whether they worked independently, and if applicable, details of automation tools used in the process. | Study quality assessment |
| Effect measures | 12 | Specify for each outcome the effect measure(s) (e.g. risk ratio, mean difference) used in the synthesis or presentation of results. | Cost and Effect measures |
| Synthesis methods | 13a | Describe the processes used to decide which studies were eligible for each synthesis (e.g. tabulating the study intervention characteristics and comparing against the planned groups for each synthesis (item #5)). | Cost and Effect measures; |
|  | 13b | Describe any methods required to prepare the data for presentation or synthesis, such as handling of missing summary statistics, or data conversions. | Cost and Effect measures |
|  | 13c | Describe any methods used to tabulate or visually display results of individual studies and syntheses. | Synthesis methods |
|  | 13d | Describe any methods used to synthesize results and provide a rationale for the choice(s). If meta-analysis was performed, describe the model(s), method(s) to identify the presence and extent of statistical heterogeneity, and software package(s) used. | Synthesis methods |
|  | 13e | Describe any methods used to explore possible causes of heterogeneity among study results (e.g. subgroup analysis, meta-regression). | Synthesis methods |
|  | 13f | Describe any sensitivity analyses conducted to assess robustness of the synthesized results. | Not applicable |
| Reporting bias assessment | 14 | Describe any methods used to assess risk of bias due to missing results in a synthesis (arising from reporting biases). | Not applicable |
| Certainty assessment | 15 | Describe any methods used to assess certainty (or confidence) in the body of evidence for an outcome. | Not applicable |
| **RESULTS** | | |  |
| Study selection | 16a | Describe the results of the search and selection process, from the number of records identified in the search to the number of studies included in the review, ideally using a flow diagram. | Study selection |
|  | 16b | Cite studies that might appear to meet the inclusion criteria, but which were excluded, and explain why they were excluded. | Study selection; Discussion |
| Study characteristics | 17 | Cite each included study and present its characteristics. | Study characteristics |
| Risk of bias in studies | 18 | Present assessments of risk of bias for each included study. | Quality assessment of studies |
| Results of individual studies | 19 | For all outcomes, present, for each study: (a) summary statistics for each group (where appropriate) and (b) an effect estimate and its precision (e.g. confidence/credible interval), ideally using structured tables or plots. | Individual Study Results; Table 3, Figure 3 |
| Results of syntheses | 20a | For each synthesis, briefly summaries the characteristics and risk of bias among contributing studies. | Individual Study Results; Types of economic evaluation; Educational domains |
|  | 20b | Present results of all statistical syntheses conducted. If meta-analysis was done, present for each the summary estimate and its precision (e.g. confidence/credible interval) and measures of statistical heterogeneity. If comparing groups, describe the direction of the effect. | Not applicable |
|  | 20c | Present results of all investigations of possible causes of heterogeneity among study results. | Not applicable |
|  | 20d | Present results of all sensitivity analyses conducted to assess the robustness of the synthesized results. | Not applicable |
| Reporting biases | 21 | Present assessments of risk of bias due to missing results (arising from reporting biases) for each synthesis assessed. | Not applicable |
| Certainty of evidence | 22 | Present assessments of certainty (or confidence) in the body of evidence for each outcome assessed. | Not applicable |
| **DISCUSSION** | | |  |
| Discussion | 23a | Provide a general interpretation of the results in the context of other evidence. | Discussion |
|  | 23b | Discuss any limitations of the evidence included in the review. | Quality of included studies |
|  | 23c | Discuss any limitations of the review processes used. | Strengths and limitations |
|  | 23d | Discuss implications of the results for practice, policy, and future research. | Conclusion |
| **OTHER INFORMATION** | | |  |
| Registration and protocol | 24a | Provide registration information for the review, including register name and registration number, or state that the review was not registered. | Protocol registration |
|  | 24b | Indicate where the review protocol can be accessed, or state that a protocol was not prepared. | Protocol registration |
|  | 24c | Describe and explain any amendments to information provided at registration or in the protocol. | Not applicable |
| Support | 25 | Describe sources of financial or non-financial support for the review, and the role of the funders or sponsors in the review. | Financial support |
| Competing interests | 26 | Declare any competing interests of review authors. | Competing interests |
| Availability of data, code and other materials | 27 | Report which of the following are publicly available and where they can be found: template data collection forms; data extracted from included studies; data used for all analyses; analytic code; any other materials used in the review. | Data availability |

Supplementary Table 4: PRISMA 2020 statement.
