# Supplementary material for: Economic evaluations in undergraduate medical education: a systematic review
Source: BMJ Open. 2025 Mar 13;15(3):e091911. doi: 10.1136/bmjopen-2024-091911 (PMC11907045; doi:10.1136/bmjopen-2024-091911)
Supplement: Supplementary file 2 [file bmjopen-2024-091911.reviewer_comments.pdf]

PEER REVIEW HISTORY

BMJ Open publishes all reviews undertaken for accepted manuscripts. Reviewers are asked to complete a checklist review form and are provided with free text boxes to elaborate on their assessment. These free text comments are reproduced below.

ARTICLE DETAILS

Title (Provisional)

Economic evaluations in undergraduate Medical Education: A systematic review

Authors

Dijk, Stijntje Willemijn; Essafi, Skander; Niehot, Christa; Wong, John B.; Hunink, Myriam; Woltman, Andrea M

VERSION 1 - REVIEW

**Reviewer**	**1**
**Name**	**Yaros, Jennifer**
**Affiliation**	**Maastricht University Faculty of Health Medicine and Life Sciences, Health Service Research**
**Date**	**27-Sep-2024**
**COI**	**None**

Dear Authors,

Thank you for your efforts to improve our understanding of the economics of medical education. Given the increased pressure on healthcare systems worldwide and the growing resource scarcity within higher education, knowledge of medical education economics is necessary to balance the forces of costs and quality and relevant to a wide audience of educational leaders and decision-makers.

Below you will find my recommendations for revisions to improve the quality of your manuscript.

1. Research Question & Study Objectives

MAJOR
In general, I found the study objectives to be clear and to the point – you seek to explore the state of economic evaluation conduct, reporting and quality of evidence in undergraduate medical education. However, I did not see an explicitly stated research question and the broader purpose of this investigation remains unclear to me. Why is

this information important to uncover, what specific added value will it offer, what is its practical significance and what is the risk of leaving this information undiscovered?

MINOR
This manuscript is an exploration limited to undergraduate medical education, yet throughout the narrative you refer to this population simply as medical education. For sake of clarity and consistency, I would maintain the term undergraduate medical education (UME) throughout the text. Furthermore, I find there is a bit of inconsistency in the choice of economic terms used in the manuscript. In the Abstract Results you state that "most studies were cost-analysis…" yet Table 1 states that 'cost analysis' is excluded from this analysis. I recommend selecting a different phrasing for the abstract to limit confusion.

Lastly, but of minor importance: I do somewhat question the inclusion of decision analytic models in this review. There are two reasons for this. First, there is a fair amount of space devoted to this topic, which only applies to a single article. Second, that article is quite different in nature from the rest of the included literature in that it explores the health of the students across their lifetime from a societal perspective, rather than exploring direct costs of education from the institution perspective, as is the case in all other articles. This being said, the article in question does meet the stated inclusion criteria and does provides an expanded perspective on the costs of medical education. Therefore, I would like to see the rationale for its inclusion strengthened, if you chose to leave it in this review or I would remove it.

2. Title/Abstract

The title is clear and accurately represents this investigation. However, if there is room to indicate the purpose of the investigation within the title, it would help direct readers to this paper and better distinguish it from other large-scale reviews, of which there are several. For example, "Economic evaluations in undergraduate medical education: A systematic review of conduct, reporting and quality of evidence" better delimits the focus of this investigation.

The abstract is a sufficient representation of the full text. Please be sure to incorporate/adjust all major revisions to the publication within the abstract, as well – with particular attention to the purpose and discussion.

3. Methods

Overall, I find this to be a comprehensive systematic literature review. Your search strategy includes a broad range of evidence sources and adheres to established reporting guidelines. However, not all necessary information for reproducibility is made explicit. For example, the inclusion/exclusion criteria are only presented in table format.

Please include a narrative summary of this information, as well. Additionally, the dates for the search strategy are missing from the main text and the supplemental file. These also need to be included. There are also some inconsistencies in the naming of some of the data items between the methods narrative and the results table (i.e. target population vs study sample, effect measure vs outcome measure, etc.). Please adjust this for easy of interpretability. Additionally, it is stated that use of economic evaluation reporting guidelines was part of the data extraction, but I do not see this reported within the results table. Lastly, please cite the exchange rate and inflation calculator used to convert costs into 2023 USD.

In addition, I feel that the synthesis of evidence can be expanded upon to indicate to the readers how information will be presented in the results.

Furthermore, there are a few methodological items that are currently presented in the results section and should be moved to the methods. For example, in the Results Study Selection it is stated that additional articles were located during the screening process, please indicate this within the methods section. Likewise, within the Results of Individual Studies: It is mentioned that not all papers explicitly state which arm of the study was the intervention and which was the comparator, along with the assumption made to classify these in the results– please move this and indicate any other assumptions made to the methods section. Finally, the definition of a decision analytic model should also be included in the methods, not the results.

4. Results

MAJOR
In general, the results reflect the research objectives though there are a few areas where greater clarity could be achieved. First, Figure 3 is described as a cost-effectiveness plane, but presented as a 3x3 table. It would be cleaner, clearer and in-keeping with economic standards to present this information in a ICER plane. I find the section on educational domains is a bit inconsistent, as it is a mixture of reporting on the types of education domains represented in the literature and sometimes, but not always, includes the cost-consequence conclusions of the original investigations. I would recommend separating these two concepts so that reporting of educational domains offers an overview of the number and percentage of publications investigating each domain and then discuss the cost-consequence conclusions of the original investigations within a separate section.

Results Table: Please review the table to ensure that all abbreviated terms are explained and that the abbreviations are consistent throughout the tables.

MINOR
In both the narrative and PRISMA table, it would be useful to give some indication of the reason why 4241 articles were excluded from the title and abstract screening.

Throughout the reporting of results, it would be helpful to include both the absolute number and percentage of each variable extracted, rather than just the absolute number.

5. Discussion & Conclusion

I found the discussion a bit difficult to follow and believe it would benefit from clarification and reorganization. The opening paragraph was a bit vague in its generality and would benefit from a more concrete summarization of the work that was carried out (we did a, b, c…), followed more succinct summary of the key findings (we found x, y, z…) and how they compare to prior evidence on this topic (and this is how they compare to other reviews…).

I was surprised to see that the inability to pool evidence was mentioned in the abstract, but not mentioned in the discussion. Given that the high-level heterogeneity impacts the quality of evidence and the ability to synthesize knowledge and advance understanding, this should appear somewhere in the discussion and conclusions and must be considered in light of your recommendations for more holistic, context-specific approaches to cost-effectiveness analysis – meaning: is this a realistic recommendation when we have yet to establish consistency in the basics of economic analyses?

Lastly, I would like to see the practical implications of failing to address the gaps in your findings made more explicit.

Supplemental Materials

Figure 2: I'm not sure I agree with the statement that "the number of published papers is relatively stable over time." From the graphical representation, there appears to be 3-fold increase in the number of publications.

**Reviewer**	**2**
**Name**	**Massou , Efthalia**
**Affiliation**	**University of Cambridge, Public Health and Primary Care**
**Date**	**20-Oct-2024**
**COI**	**None**

Dear Authors,

As a reviewer of yourmanuscript, titled "Economic Evaluations in Undergraduate Medical Education: A Systematic Review," I found several strengths as well as areas that require

further improvement. First, in terms of the strengths, the study addresses an underexplored area in medical education—economic evaluations—which is timely and relevant given the rising costs of education. By focusing on cost-effectiveness in undergraduate medical education, the manuscript fills a gap in the literature. The systematic review adheres to PRISMA guidelines, which strengthens the transparency and reproducibility of the study. The use of multiple databases (Medline, Embase, Web of Science, etc.) and a clear search strategy adds robustness. The application of the CHEQUE tool to evaluate the methodological quality of the included studies is appropriate, providing readers with a structured assessment of economic evaluations in medical education. I really liked the visual representations like Figure 2 which are helpful in conveying this information clearly. Finally, the discussion highlights the limitations of current economic evaluations in medical education, particularly the narrow scope (e.g., payer perspective, lack of long-term effects). The recommendations for future research are thoughtful and grounded in the review's findings.

Now, in terms of the areas for improvement, I noticed that the manuscript occasionally assumes a high level of familiarity with health economics concepts (e.g., ICER, CHEQUE, and WTP) without providing sufficient explanation for general readers. Defining these key terms in the introduction or methods section would enhance readability. Also, while the paper synthesizes findings across studies, there is a lack of detailed explanation of the heterogeneity in the included studies. More explicit discussion of why certain types of studies (e.g., cost-utility analysis vs. cost-benefit analysis) may be more or less applicable to medical education contexts could add depth. The results could benefit from more detailed breakdowns of trends in cost-effectiveness findings across different educational domains (e.g., practical skills training vs. general instructional approaches). A table summarizing key differences might enhance clarity. I also noticed some gaps in methodological transparency. More specifically, the manuscript mentions converting costs into 2023 USD but does not detail the process used (e.g., specific inflation rates or exchange rates). Including this information would improve transparency. Some methodological decisions (e.g., not contacting subject experts, not browsing unindexed journals) may also limit the comprehensiveness of the review. Briefly justifying these decisions in the methods section would clarify their rationale. The manuscript should ensure consistent use of terms, especially when referring to different types of economic evaluations (e.g., cost-effectiveness analysis, cost-minimization analysis, etc.). Some terms are used interchangeably, which could confuse readers unfamiliar with economic evaluation distinctions.

Finally, the conclusion, while comprehensive, could better emphasize the real-world implications of the findings. For instance, how might the lack of comprehensive cost-effectiveness studies in medical education impact policy decisions? Offering more actionable recommendations for educators and policymakers would strengthen the manuscript's impact.

VERSION 1 - AUTHOR RESPONSE

Comments from reviewer 1:

Dr. Jennifer Yaros, Maastricht University Faculty of Health Medicine and Life Sciences

Comments to the Author:

Dear Authors,

Thank you for your efforts to improve our understanding of the economics of medical education. Given the increased pressure on healthcare systems worldwide and the growing resource scarcity within higher education, knowledge of medical education economics is necessary to balance the forces of costs and quality and relevant to a wide audience of educational leaders and decision-makers.

Below you will find my recommendations for revisions to improve the quality of your manuscript.

R1.1 In general, I found the study objectives to be clear and to the point – you seek to explore the state of economic evaluation conduct, reporting and quality of evidence in undergraduate medical education. I did not see an explicitly stated research question and the broader purpose of this investigation remains unclear to me. Why is this information important to uncover, what specific added value will it offer, what is its practical significance and what is the risk of leaving this information undiscovered?

Response: We appreciate the reviewer's feedback on the clarity of the research question. In this paper we answer the question "How are economic evaluations conducted and reported in the field of undergraduate medical education, and what are the implications of these findings for the future of medical education and research?". This question had been posted in the abstract and has now been added more explicitly to the main manuscript. We have also specifically added in secondary aims to further clarify what our paper investigates. The broader purpose was firstly to identify the current state of economic evaluations in medical education, to identify areas of best practice and areas for improvement. Secondly to assess the quality of such previously conducted evaluations to get an estimate of the reliability of previous findings. Thirdly, and perhaps most importantly given the limited number of full evaluations we found; the paper highlights the importance of economic evaluation in medical education by demonstrating its value in making informed decisions about resource allocation. We hope that our review can help to encourage greater adoption of methods frequently applied in health economics, including decision modelling techniques to which we have dedicated a specific section to in our manuscript. Finally, we hope that our review can help to inform future research by showcasing best and worst practices, and we hope that it can ultimately contribute to what evidence is gathered and in which manner to inform policy. In the paper, we show examples of how neglecting costs can lead to decisions that share similar effectiveness but widely differential costs, highlighting how resources can be used elsewhere. By addressing these objectives, we aim to contribute to a more efficient medical education system that maximizes resource allocation.

We have added a statement on the consequence of neglecting costs to the introduction (and, in response to a later comment, the discussion), and added the specific research question and secondary aims to the Aim paragraph.

R1.2 This manuscript is an exploration limited to undergraduate medical education, yet throughout the narrative you refer to this population simply as medical education. For sake of clarity and consistency, I would maintain the term undergraduate medical education (UME) throughout the text.

Response: The reviewer is correct that indeed our review only included papers on undergraduate medical education. We now refer to UME in the majority of text areas, specifically those that reflect on our scope or findings. Some statements were applicable to the broader scope of medical education which we have kept as medical education, and we did not add the abbreviation to the abstract.

R1.3 I find there is a bit of inconsistency in the choice of economic terms used in the manuscript. In the Abstract Results you state that "most studies were cost-analysis…" yet Table 1 states that 'cost analysis' is excluded from this analysis. I recommend selecting a different phrasing for the abstract to limit confusion.

Response: The term referred to in the abstract refers to papers that were cost-analyses performed alongside trials, meaning that within the paper they do report on both costs and effects. We do however understand the confusion and have adjusted this to lists of costs presented alongside effectiveness trials.

R1.4 I do somewhat question the inclusion of decision analytic models in this review. There are two reasons for this. First, there is a fair amount of space devoted to this topic, which only applies to a single article. Second, that article is quite different in nature from the rest of the included literature in that it explores the health of the students across their lifetime from a societal perspective, rather than exploring direct costs of education from the institution perspective, as is the case in all other articles. This being said, the article in question does meet the stated inclusion criteria and does provides an expanded perspective on the costs of medical education. Therefore, I would like to see the rationale for its inclusion strengthened, if you chose to leave it in this review or I would remove it.

Response: We appreciate the reviewer's comment regarding the inclusion of decision analytic models. While this topic is discussed in detail, it's true that only one study was identified. However, we believe that decision analytic modeling is a powerful tool for evaluating the long-term costs and benefits of medical education interventions. By incorporating this approach, researchers can account for factors like student health, future career paths, and societal impact, which may not be captured in cost-effectiveness analyses applied more frequently within medical education. This type of modeling is however a very common best practice analysis tool within the field of health economic evaluations. Dedicating additional focus to this area of research even though (or perhaps especially because) only one paper used this design was a conscious decision by the authors to highlight the methods behind this research to hopefully inspire future research in this area. We have shifted the location in which we address decision analytic modeling to the discussion to highlight how we believe they are a potential answer to overcoming the gaps in the UME literature.

The specific study included in our review reports an intervention that was presented to all medical students within a medical school with the purpose of improving their health as students and future healthcare professionals, although we had within our research team discussed its main focus on their health professional role. It however did formally meet our inclusion criterion of an intervention within the medical school. While the majority of studies in our review focused on institutional perspectives, we believe that a societal perspective is equally important and the perspective (institutional, healthcare, societal, payer or other) was not an explicit exclusion criterion in our study. The Second Panel on Cost-Effectiveness in Health and Medicine actually recommends always considering a societal perspective in economic evaluations. We have no grounds to exclude this paper from our study based

on our set exclusion and inclusion criteria. We have therefore not adjusted the selection of included papers based on this comment.

R1.5 The title is clear and accurately represents this investigation. However, if there is room to indicate the purpose of the investigation within the title, it would help direct readers to this paper and better distinguish it from other large-scale reviews, of which there are several. For example, "Economic evaluations in undergraduate medical education: A systematic review of conduct, reporting and quality of evidence" better delimits the focus of this investigation.

Response: We thank the reviewer for this suggestion. We have sincerely considered and discussed the comment within our research team. Ultimately, we agree with the reviewer that the suggested title would more accurately reflect the primary focus of the investigation, we in the end felt that the overall scope and content of the paper was broader than conduct, reporting and quality of evidence, as it also considers the outcomes of the included reviews, and the investigations themselves, which was one of the reasons for us to not present the review as a scoping review rather than systematic review. We have therefore decided not to change the manuscript title.

R1.6 The abstract is a sufficient representation of the full text. Please be sure to incorporate/adjust all major revisions to the publication within the abstract, as well – with particular attention to the purpose and discussion.

Response: We have updated our abstract based on the revised manuscript.

R1.7 I find this to be a comprehensive systematic literature review. Your search strategy includes a broad range of evidence sources and adheres to established reporting guidelines. However, not all necessary information for reproducibility is made explicit. For example, the inclusion/exclusion criteria are only presented in table format. Please include a narrative summary of this information, as well.

Response: We thank the reviewer for their comment and have added a narrative summary of the information from Table 1 in-text.

R1.8 the dates for the search strategy are missing from the main text and the supplemental file. These also need to be included. There are also some inconsistencies in the naming of some of the data items between the methods narrative and the results table (i.e. target population vs study sample, effect measure vs outcome measure, etc.). Please adjust this for easy of interpretability.

The reviewer is correct that the headings we listed in-text for the data extraction sheet for target population and effect measure were not identical to the headings used in the tables. We have now listed these as study sample and outcome measure.

R1.9 Additionally, it is stated that use of economic evaluation reporting guidelines was part of the data extraction, but I do not see this reported within the results table. Lastly, please cite the exchange rate and inflation calculator used to convert costs into 2023 USD.

We have added a reference to the exchange rate and inflation calculator to the manuscript. Initially, we added the column requested by the reviewer to our revision. However, we have been requested by the BMJ Open technical editors to constrict the tables to max 2 pages and max 8 columns each. We have now removed this column again from the table in the main manuscript and added an extended table to the supplementary materials that includes the requested column.

R1.10 Furthermore, there are a few methodological items that are currently presented in the results section and should be moved to the methods. For example, in the Results Study Selection it is stated that additional articles were located during the screening process, please indicate this within the methods section. Likewise, within the Results of Individual Studies: It is mentioned that not all papers explicitly state which arm of the study was the intervention and which was the comparator, along with the assumption made to classify these in the results– please move this and indicate any other assumptions made to the methods section. Finally, the definition of a decision analytic model should also be included in the methods, not the results.

Response: We thank the reviewer for these considerations, and we agree for most of the suggested items. We included a statement in the methods that we consulted relevant identified systematic reviews by our search for additional citations that met our inclusion criteria. The identification of these three items is still under the results, as these were identified as a result of our search. We also moved the statement on intervention ambiguity to the methods section. We still mention in the results that not all papers explicitly mention which strategy is the intervention and which is the comparator, as this also reflects on our study findings that CEAs were poorly reported. We moved the description of decision analytic models to the methods, and the example of questions that could potentially be answered by such models to the discussion.

R1.11 I feel that the synthesis of evidence can be expanded upon to indicate to the readers how information will be presented in the results.

Response: We have expanded the synthesis section of our methods.

R1.12 In general, the results reflect the research objectives though there are a few areas where greater clarity could be achieved. First, Figure 3 is described as a cost-effectiveness plane, but presented as a 3x3 table. It would be cleaner, clearer and in-keeping with economic standards to present this information in a ICER plane.

Response: We thank the reviewer for their comment. To convert the information to a true CE-plane we would require information/standardization of the outcome measure that would allow us to place the different studies at a relative distance to the null. As these interventions are not being compared to each other, we think that such standardization would not provide additional information to the reader. This is the reason why we had labeled Figure 3 as a "*Descriptive* CE-plane". We can see however that this term may seem confusing to the reader as we have removed the cross at the 0 lines to improve readability. We have adjusted the figure and figure name to better reflect our original intention.

R1.13 I find the section on educational domains is a bit inconsistent, as it is a mixture of reporting on the types of education domains represented in the literature and sometimes, but not always, includes the cost-consequence conclusions of the original investigations. I would recommend separating these two concepts so that reporting of educational domains offers an overview of the number and percentage of publications investigating each domain and then discuss the cost-consequence conclusions of the original investigations within a separate section.

Response: We thank the reviewer for their comment. In the section on educational domains we have attempted to first give an impression of which domains were addressed within our review (first paragraph), then dedicate one paragraph for each of these domains to showcase domain specific characteristics of economic evaluations, mostly which types of costs and which effects were included, and which comparisons we made. The content of these paragraphs is distinguished from cost-effectiveness considerations that are presented in the "Individual study results" section. We have now

highlighted the domain addressed in each paragraph more clearly to make it easier for readers to see which UME domain is addressed.

R1.14 Results Table: Please review the table to ensure that all abbreviated terms are explained and that the abbreviations are consistent throughout the tables.

Response: We thank the reviewer for remarking this discrepancy and have added missing abbreviations and resolved differences between tables 2 and 3.

R1.15 In both the narrative and PRISMA table, it would be useful to give some indication of the reason why 4241 articles were excluded from the title and abstract screening. Throughout the reporting of results, it would be helpful to include both the absolute number and percentage of each variable extracted, rather than just the absolute number.

Response: Our review narrative describes the reasons why full-text papers were excluded as presented under study selection, in line with the PRISMA guidelines. However, at the title abstract screening stage we did not document the reasons why each paper was excluded, as this is not required and significantly slows the screening process. As a general impression, we can share that in a vast number of cases, papers were excluded because they either concerned teaching students about cost-effectiveness, were commentaries on the need to consider cost-effectiveness in medical education, or were cost analyses stating for example the costs of training one medical students incurred by a specific university.

We have included the percentage in addition to the absolute number for the items addressed within the results section.

R1.16 I found the discussion a bit difficult to follow and believe it would benefit from clarification and reorganization. The opening paragraph was a bit vague in its generality and would benefit from a more concrete summarization of the work that was carried out (we did a, b, c…), followed more succinct summary of the key findings (we found x, y, z…) and how they compare to prior evidence on this topic (and this is how they compare to other reviews…).

Response: We have revised the discussion to enhance clarity and organization. The opening paragraph now provides a brief overview of our work, highlighting the key gaps identified in our review and the consequences of not addressing them. Following this, we include a dedicated section emphasizing the importance of addressing these gaps. The discussion is then structured around specific findings from our review, with each paragraph focusing on one key issue: the choice and level of outcomes evaluated in the included studies, the scope and quality of the studies, their handling of uncertainty, and how these gaps could be addressed through methods like decision-analytic modeling. Additionally, we have refined the opening paragraph to introduce these points. We do note within this paragraph that no prior reviews have comprehensively addressed both costs and effects in this context. Later in the discussion, we reference related reviews that address different aspects of the topic, such as Yaros et al.'s review on cost items in medical education and previous guidance on conducting economic evaluations in medical education. However, we note that these works, including the book on cost-effectiveness in medical education, do not provide a compilation of cost-effectiveness analyses in the field.

R1.17 I was surprised to see that the inability to pool evidence was mentioned in the abstract, but not mentioned in the discussion. Given that the high-level heterogeneity impacts the quality of evidence and the ability to synthesize knowledge and advance understanding, this should appear somewhere in the discussion and conclusions and must be considered in light of your recommendations for more

holistic, context-specific approaches to cost-effectiveness analysis – meaning: is this a realistic recommendation when we have yet to establish consistency in the basics of economic analyses?

Response: We thank the reviewer for highlighting the discrepancy between the abstract and discussion regarding study heterogeneity. While we did not conduct a meta-analysis or pool evidence due to the diverse nature of the studies and the diverse nature of study outcomes, we acknowledge that this was not the primary focus of our study. Our primary objective was to explore the methodologies and practices of economic evaluations within undergraduate medical education. Given the reviewer's input, we have removed the reference to study heterogeneity from the abstract to avoid any potential confusion.

R1.18 Lastly, I would like to see the practical implications of failing to address the gaps in your findings made more explicit.

Response: We have now dedicated a section of the discussion to discuss the consequences of not addressing these gaps in literature, specifically the consideration of cost-effectiveness and uncertainty, the finite resources we have available, and the social accountability of medical schools.

R1.19 Supplemental Materials

Figure 2: I'm not sure I agree with the statement that "the number of published papers is relatively stable over time." From the graphical representation, there appears to be 3-fold increase in the number of publications.

Response: The figure represents the cumulative number of papers published over time. While there is a 3-fold increase in publications, we believe the increase in publications is relatively stable over time, rather than for example showing an exponential growth or halt. We have adjusted the wording of this statement to reflect this intention ("the increase in publications is relatively stable")

<u>Comments from Reviewer 2</u>

<u>Reviewer: 2 Dr. Efthalia Massou , University of Cambridge</u>

<u>Comments to the Author:</u>

R2.1 the study addresses an underexplored area in medical education—economic evaluations—which is timely and relevant given the rising costs of education. By focusing on cost-effectiveness in undergraduate medical education, the manuscript fills a gap in the literature. The systematic review adheres to PRISMA guidelines, which strengthens the transparency and reproducibility of the study. The use of multiple databases (Medline, Embase, Web of Science, etc.) and a clear search strategy adds robustness.

Response: We thank the reviewer for their comment.

R2.2 The application of the CHEQUE tool to evaluate the methodological quality of the included studies is appropriate, providing readers with a structured assessment of economic evaluations in medical education. I really liked the visual representations like Figure 2 which are helpful in conveying this information clearly.

Response: We thank the reviewer for their comment, and particularly appreciate receiving feedback on this CHEQUE figure as this review was the first to apply this new quality assessment tool in a systematic review setting. We are happy to share that we have written a separate methodological paper on the experience of using CHEQUE and have shared all R code that created Figure 2 in a public repository.

R2.3 The discussion highlights the limitations of current economic evaluations in medical education, particularly the narrow scope (e.g., payer perspective, lack of long-term effects). The recommendations for future research are thoughtful and grounded in the review's findings.

Response: We thank the reviewer for their comment.

R2.4 I noticed that the manuscript occasionally assumes a high level of familiarity with health economics concepts (e.g., ICER, CHEQUE, and WTP) without providing sufficient explanation for general readers. Defining these key terms in the introduction or methods section would enhance readability.

Response: The reviewer is correct that some familiarity was assumed for this review. We had indeed within the research team discussed the balance between providing sufficient explanation while considering the word limit and not transforming the review into a tutorial and instead refer to existing guidance. Based on this reviewer comment we have now added a separate box prior to at the start of the paper that provides an overview of key concepts so that more proficient readers can skip this additional information, while more novel readers receive the context they require.

R2.5 while the paper synthesizes findings across studies, there is a lack of detailed explanation of the heterogeneity in the included studies. More explicit discussion of why certain types of studies (e.g., cost-utility analysis vs. cost-benefit analysis) may be more or less applicable to medical education contexts could add depth.

Response: We thank the reviewer for their comment. Within our author group, we do not think that necessarily one type of analysis would be more appropriate than another within medical education context, but rather that this type of analysis depends on the research question. However, what we do see throughout the collected papers that the type of economic evaluation seems to be given little thought up front, and rarely consider high level or long-term consequences, or uncertainty, which we address within our discussion section. We have now reiterated the need for comprehensive economic evaluations in the discussion, and have added that we do not believe one type of analysis is better than others without considering the research question to the discussion. We have also moved the table with examples of decision analytic models that could be applied within medical education research and could address both these points to the discussion section.

R2.6 The results could benefit from more detailed breakdowns of trends in cost-effectiveness findings across different educational domains (e.g., practical skills training vs. general instructional approaches). A table summarizing key differences might enhance clarity. I also noticed some gaps in methodological transparency.

Response: We expanded our methods section to provide further details in response to both reviewers' comments. If there are particular areas where the reviewer believes methodological transparency is missing, we of course remain available to add further details.

R2.7 the manuscript mentions converting costs into 2023 USD but does not detail the process used (e.g., specific inflation rates or exchange rates). Including this information would improve transparency.

Response: We have added a reference to the exchange rate and inflation calculator to the manuscript.

R2.8 Some methodological decisions (e.g., not contacting subject experts, not browsing unindexed journals) may also limit the comprehensiveness of the review. Briefly justifying these decisions in the methods section would clarify their rationale.

Response: Our review includes the 200 highest-ranked references from Google Scholar, a method employed by Bramer et al. (2018). This approach allows us to capture a broad range of relevant literature, including potentially valuable insights from unindexed journals. Additionally, our research team includes a mixture of health economists, decision scientists, methodologists, clinicians, medical educationalists and information specialists who all contributed their individual expertise to the paper. The statement in the methods section specifically refers to the search strategy, where in non-systematic reviews asking experts for preferred papers or browsing through unindexed journals could be considered as additional methods of retrieving potential citations. Due to the thoroughness of the search itself, we believe that our search strategy is considered a strength, rather than a limitation. To avoid confusion with the audience from this elaboration on non-systematic additional approaches we could have taken but didn't, we have removed this statement from the methods section.
If there are particular areas where the reviewer believes additional justification or exploration is warranted, we are happy to provide further details or conduct additional analysis.

R2.9 The manuscript should ensure consistent use of terms, especially when referring to different types of economic evaluations (e.g., cost-effectiveness analysis, cost-minimization analysis, etc.). Some terms are used interchangeably, which could confuse readers unfamiliar with economic evaluation distinctions.

Response: We hope the new table we created in response to comment R2.4 will help to more clearly distinguish the relevant terminology used in our paper.

R2.10 Finally, the conclusion, while comprehensive, could better emphasize the real-world implications of the findings. For instance, how might the lack of comprehensive cost-effectiveness studies in medical education impact policy decisions? Offering more actionable recommendations for educators and policymakers would strengthen the manuscript's impact.

Response: We thank the reviewer for this thoughtful comment. We have in the revision of our manuscript further emphasized the consequences of overlooking cost-effectiveness within undergraduate medical education.

VERSION 2 - REVIEW

Reviewer 1

Name Yaros, Jennifer

Affiliation Maastricht University Faculty of Health Medicine and Life Sciences, Health Service Research

Date 24-Dec-2024

COI

Dear Authors,

Thank you for your efforts to improve the quality of this manuscript. I accept the major revisions you have provided as sufficient. However, I do feel that there are some minor revisions which should be addressed prior to publication.

Title/Abstract

I accept the authors' reasoning to maintain the original title as is. However, I still feel that the abstract background should mention that quality assessment was one of the aims of the investigation as it is referred to within the abstract methods, results and discussion.

Introduction / Discussion / Conclusion

In the Introduction, Discussion and Conclusion, you refer to the "full body of literature" as previously unexplored. I find this phrasing a bit misleading and feel you must indicate that you are referring to the body of undergraduate medical education literature and not all medical education literature. This applies to:

• Introduction, second to last paragraph, first sentence

• Discussion, second paragraph, second sentence

• Conclusion, first paragraph, first sentence only

And additionally applies to:

• Discussion, fourth paragraph, third sentence (where you refer to the scope of your investigation)

Results

Thank you for including the use of economic evaluation reporting guidelines in the results table in the supplemental file and addressing inconsistencies in the naming convention in the results table and supplemental file. However, under the following column headings "WTP Threshold" and "Sensitivity Analysis" in the Results Table and "Economic Evaluation Reporting Guideline" in the Supplemental File, you use the abbreviation N/R which is not defined below the tables. Please rectify this so it is clear to readers what this term means.

Discussion

I feel the strengths of this study require further development. First, I would expand beyond your methodological strengths. From my perspective one of the primary strengths of this study is the comprehensive review of economic evaluations and its specificity of topic (undergraduate medical education), which contribute to the building of knowledge on a previously unexplored topic. Second, the application of the CHEQUE tool is certainly a strength but requires at least a single sentence of rationale to explain why this is a strength. Lastly, I am not convinced by the rationale you have provided for your third strength: "The two papers[50,61] identified by a related systematic review and that were also eligible according to our inclusion criteria, were also identified by our review, supporting the

thoroughness of our search," as I do not see how two papers in common with another systematic review equates to thoroughness. Please note, I do not question the thoroughness of your search, just the rationale you have provided for it. If you'd like to leave this strength in, I recommend highlighting other aspects of thoroughness (i.e. consultation with an information specialists, use of multiple data sources, and articles reviewed in duplicate).

**Reviewer**	**2**
**Name**	**Massou , Efthalia**
**Affiliation**	**University of Cambridge, Public Health and Primary Care**
**Date**	**21-Dec-2024**
**COI**	

Thank you for addressing so thoroughly the comments.

VERSION 2 - AUTHOR RESPONSE

Comments from reviewer 1:
Dr. Jennifer Yaros, Maastricht University Faculty of Health Medicine and Life Sciences
Comments to the Author:
Dear Authors,

Thank you for your efforts to improve the quality of this manuscript. I accept the major revisions you have provided as sufficient. However, I do feel that there are some minor revisions which should be addressed prior to publication.

R1.1 Title/Abstract: I accept the authors' reasoning to maintain the original title as is. However, I still feel that the abstract background should mention that quality assessment was one of the aims of the investigation as it is referred to within the abstract methods, results and discussion.

Response: We thank the reviewer for their comment. We have added the objective of assessing the quality of published economic evaluations to the abstract background section.
R1.2 Introduction / Discussion / Conclusion: In the Introduction, Discussion and Conclusion, you refer to the "full body of literature" as previously unexplored. I find this phrasing a bit misleading and feel you must indicate that you are referring to the body of undergraduate medical education literature and not all medical education literature. This applies to:
• Introduction, second to last paragraph, first sentence
• Discussion, second paragraph, second sentence
• Conclusion, first paragraph, first sentence only

And additionally applies to:
• Discussion, fourth paragraph, third sentence (where you refer to the scope of your investigation)
Response: We thank the reviewer for their comment. We have added the statement of "UME" to the body of literature statements referred to by the reviewer, with the exception of the introduction, as this statement refers to background information that this has not been explored in medical education,

UME or otherwise, and the discussion fourth paragraph, as this refers to a general statement that research objectives should be aligned with methods, which we do not consider limited to our study scope of undergraduate medical education.
R1.3 Results: Thank you for including the use of economic evaluation reporting guidelines in the results table in the supplemental file and addressing inconsistencies in the naming convention in the results table and supplemental file. However, under the following column headings "WTP Threshold" and "Sensitivity Analysis" in the Results Table and "Economic Evaluation Reporting Guideline" in the Supplemental File, you use the abbreviation N/R which is not defined below the tables. Please rectify this so it is clear to readers what this term means.
Response: We thank the reviewer for their diligence, and have added the explanation N/R = Not reported to the relevant descriptions.
R1.4 Discussion: I feel the strengths of this study require further development. First, I would expand beyond your methodological strengths. From my perspective one of the primary strengths of this study is the comprehensive review of economic evaluations and its specificity of topic (undergraduate medical education), which contribute to the building of knowledge on a previously unexplored topic. Second, the application of the CHEQUE tool is certainly a strength but requires at least a single sentence of rationale to explain why this is a strength. Lastly, I am not convinced by the rationale you have provided for your third strength: "The two papers[50,61] identified by a related systematic review and that were also eligible according to our inclusion criteria, were also identified by our review, supporting the thoroughness of our search," as I do not see how two papers in common with another systematic review equates to thoroughness. Please note, I do not question the thoroughness of your search, just the rationale you have provided for it. If you'd like to leave this strength in, I recommend highlighting other aspects of thoroughness (i.e. consultation with an information specialists, use of multiple data sources, and articles reviewed in duplicate).
Response: We thank the reviewer for this critical feedback as well as their reflection on the strengths of our paper. We have adjusted the manuscript accordingly. With respect to our reasoning for thoroughness, we believe that the statement regarding the manuscript identification does support our conclusion. Specifically, the statement demonstrates that our search strategy successfully identified all eligible papers from two previous systematic reviews. This indicates that our search did not miss any papers included in those reviews.

Conversely, if another review had included 10 eligible papers that our search did not identify, it would suggest that our search lacked certain elements covered by their search strategy. Therefore, we have retained this statement in the manuscript as it provides valuable evidence of our search's comprehensiveness.
With respect to our reasoning for thoroughness, we believe that the statement supports this by verifying that within the papers that were included in previous systematic reviews, all (albeit only 2) papers that would have also been eligible within our review were also identified, meaning that their search did not result in papers that we missed. If the other review had included 10 papers that would have also been eligible according to our eligibility criteria, it means that our search lacked elements that their search did cover. We have therefore left this statement within the manuscript.

Comments from Reviewer 2
Reviewer: 2 Dr. Efthalia Massou , University of Cambridge
Comments to the Author:
R2.1 Thank you for addressing so thoroughly the comments.
Response: We thank the reviewer for their comment and their time during the review process
